# Identifying lead hits in catalyst discovery by screening and deconvoluting complex mixtures of catalyst components†
†Electronic supplementary information (ESI) available: Experimental procedures and spectral data. See DOI: 10.1039/c5sc00268k



**DOI:** 10.1039/c5sc00268k

**Published:** 2015-02-16

**Authors:** Eléna Wolf, Edward Richmond, Joseph Moran

**Affiliations:** a ISIS & icFRC , Université de Strasbourg & CNRS , 8 allée Gaspard Monge , 67000 Strasbourg , France . Email: moran@unistra.fr

## Abstract

A reaction-economic combinatorial strategy is described for lead hit identification in catalyst discovery efforts directed towards a specific transformation. Complex mixtures of rationally chosen precatalysts and ligands are screened against various reaction parameters to identify lead conditions in a small number of reactions. Iterative deconvolution of the resulting hits identifies which components contribute to the lead *in situ* generated catalyst. Application of this strategy rapidly uncovered a new mild *in situ* generated catalyst for the dehydrative Friedel–Crafts reaction as well as conditions for selective monoarylation in catalytic *ortho*-C–H arylation of unsubstituted *N*-(quinolin-8-yl)benzamide.

## Abstract 2

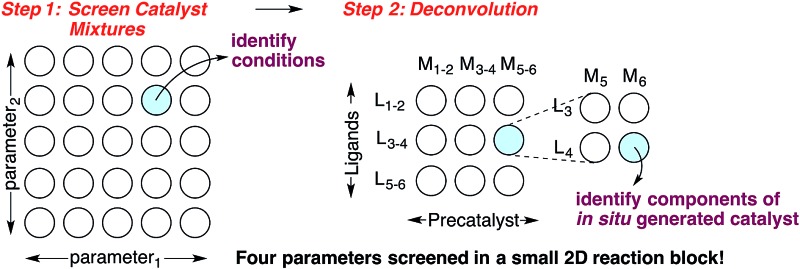
A combinatorial screening strategy is described that exploits complex mixtures of precatalysts and ligands to rapidly uncover lead *in situ* generated catalysts.

## Introduction

**Scheme 1:**
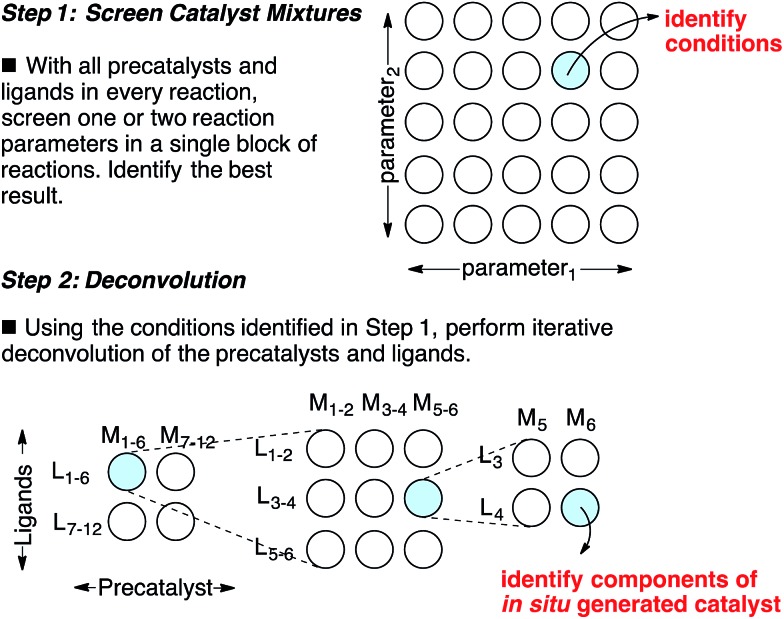
A combinatorial strategy for identifying lead “hits” in catalyst discovery by screening complex mixtures of catalyst components.

Catalyst discovery is a complex multidimensional problem that often requires extensive experimentation to obtain a lead result. In a representative catalyst discovery scenario, precatalyst, ligand, solvent, acid/base additive or temperature may all be critical reaction parameters, and incorrect choice of any one of these may result in failure to observe an initial “hit” for the desired product. In response to this reality, many creative screening approaches have been developed for catalyst discovery, each with different purposes, strengths and weaknesses.^1–4^ Of those screening approaches that are directed towards discovering a specific new catalytic transformation or a new class of catalysts for an existing transformation, few are highly general and several require laborious synthesis of labeled starting materials. Though advances in analytical technology are decreasing the barriers to general reaction development approaches using high throughput experimentation,^5^ the large number of reactions necessary to thoroughly explore the intersection of just three or four reaction parameters might still deter chemists who do not have access to high throughput reactionware and instrumentation. Complementary approaches that can reduce the number of reactions required to obtain a lead result for a specific transformation and that do not require high throughput instrumentation are appealing. Towards this goal, we have devised an approach for the identification of lead results in catalyst discovery based on the assumption that precatalysts and ligands can be screened as mixtures and later deconvoluted.^6^ By employing a complex mixture of all precatalysts and ligands in every reaction, one or two additional reaction parameters (*e.g.* solvent, acid/base, *etc.*) are screened to identify a promising result. In this way, up to four reaction parameters can be surveyed at once in a single small block of reactions, effectively ‘front-loading’ the problem of catalyst discovery (Scheme 1, Step 1). The precatalysts and ligands that contribute most to catalysis are identified by iterative deconvolution of the mixture in a manner similar to that described by Breit for mixtures of self-assembling ligands (Scheme 1, Step 2).^4^ As the aim of this approach is to accelerate catalyst discovery, the primary goal is simply to detect product formation. Thus, the fact that different precatalyst/ligand combinations exhibit different binding constants only leads to false negatives if a component critical to catalysis is completely inhibited. Risks of encountering such catalyst poisoning scenarios can be rationally minimized by tuning the ratio of precatalysts to ligands during screening. Given these considerations, we anticipated that this screening approach could be useful for reactions that involve significant molecular recognition between catalyst and substrate. Herein, we describe the successful application of this strategy to developing new catalysts for two completely different catalytic reactions. In the first example, a Friedel–Crafts reaction is used to discover a new powerful boron catalyst that arises by covalent assembly from a complex mixture of boronic acids and bidentate *O*-ligands. In a second example, application of the strategy has uncovered a catalytic system for selective mono *ortho*-C–H arylation of *N*-(quinolin-8-yl)benzamide in the absence of blocking groups at the *ortho* or *meta* positions. Our collective results indicate that a rationally guided screening approach using complex mixtures of catalyst components, while not necessarily an analytical solution to a catalyst discovery problem, is a useful complementary strategy to rapidly identifying lead “hits” in a small number of reactions without the use of high throughput equipment.

## Results and discussion

**Fig. 1:**
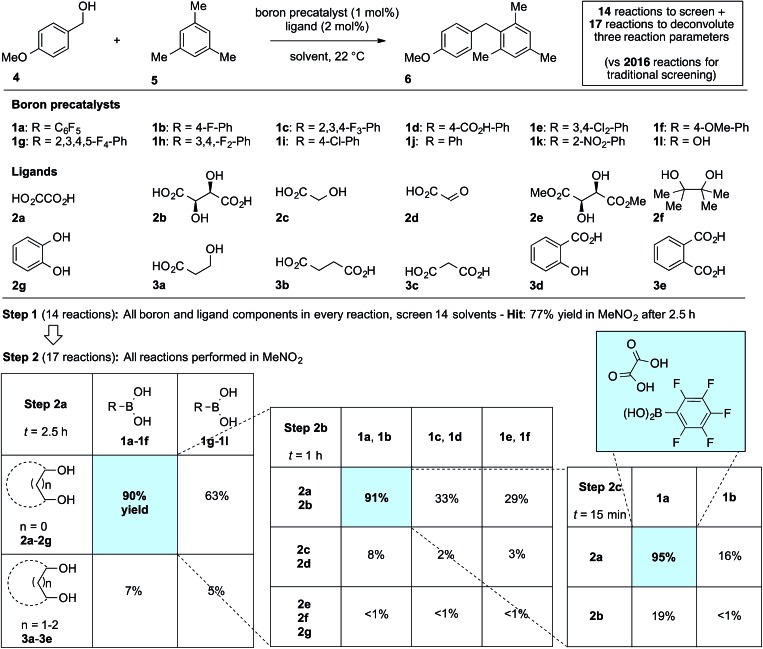
Combinatorial discovery and deconvolution of *in situ*-generated boron catalysts for the dehydrative Friedel–Crafts reaction. Conditions: 1.0 equiv. **4** (0.2 M), 3.0 equiv. **5**. Yield determined by ^1^H NMR in CDCl_3_ with DMSO (1 equiv.) as internal standard.

We first applied the catalyst discovery strategy to the selection of an *in situ* generated boron catalyst from a complex mixture of boronic acids and bidentate *O*-ligands.^7,8^ The dehydrative Friedel–Crafts reaction^9^ between *p*-methoxybenzyl alcohol (**4**) and mesitylene (**5**) to give diarylmethane (**6**) was chosen as a target reaction since it can be triggered by strong Brønsted or Lewis acids but is not enabled by boronic acids^10^ or carboxylic acids. Twelve boron precatalysts were chosen for screening, including eleven electronically diverse boronic acids and boric acid. Twelve bidentate *O*-ligands were chosen with the goal of maximizing their structural diversity,^11^ including diols, catechols, hydroxyacids and diacids. Screening a mixture of all boron precatalysts (1 mol% each) and all *O*-ligands (2 mol% each) against 14 different solvents at 22 °C resulted in 77% yield of **6** in MeNO_2_, 26% in MeCN, 3% in CH_2_Cl_2_ or DCE and <1% in the ten other solvents after 2.5 h (Fig. 1, Step 1). Thus, a lead result was identified in a three-dimensional screen requiring just 14 reactions, though the identities of the most active components were not yet known. Control experiments showed negligible reactivity when boronic acids alone or *O*-ligands alone were employed as catalysts. To facilitate the deconvolution process in MeNO_2_, the boron compounds were arbitrarily divided into two groups (**1a–1f** and **1g–1l**). The *O*-ligands were also divided into two groups (**2a–2g** and **3a–3e**). The four possible combinations of groups were screened with 1 mol% of each boron precatalyst and 2 mol% of each *O*-ligand present in the reaction at the same time (Fig. 1, Step 2a). The best result was found to come from the mixture of boron precatalysts **1a–1f** and *O*-ligands **2a–2g**, which went to completion after 2.5 h. The winning boron precatalysts were broken up into three arbitrary groups, **1a–1b**, **1c–1d** and **1e–1f**. Likewise, the winning *O*-ligands were divided into three groups, **2a–2b**, **2c–2d** and **2e–2g**. The most rapid of the nine reactions was that containing a mixture of **1a–1b** and **2a–2b**, which went to completion after 1 h (Fig. 1, Step 2b). The remaining four combinations were tested individually. Impressively, mixing 1 mol% of **1a** with 2 mol% of **2a** led to 95% yield of **6** after 15 min at room temperature (Fig. 1, Step 2c). As expected, the time for reaction completion decreased with each deconvolution step as the optimal catalyst is present in higher concentration. Only 31 reactions were required to screen and deconvolute three reaction parameters, with eventual success foreshadowed in Step 1 by a block of just 14 reactions. In contrast, screening the same three reaction parameters one at a time would require 2016 reactions, with no indication of success at the outset. Indeed, traditional linear screening arrives at the same result (see ESI†). Remarkably, the catalytic effect of **1a** + **2a** is faster than benchmark boron catalysts BF_3_·THF and B(C_6_F_5_)_3_·H_2_O,^12^ which gave 80% and 20% yield, respectively, with the remainder being starting materials under identical conditions and time. In contrast, addition of 1 mol% **1a** or 2 mol% **2a** in isolation led to <5% product after 4 h in both cases. Though the covalent assembly of boronic acids with oxalic acid to give dioxaborolanediones is known,^13,14^ catalysis by the combination of those components or by the resulting covalent adduct has not been reported.^15^

**Fig. 2:**
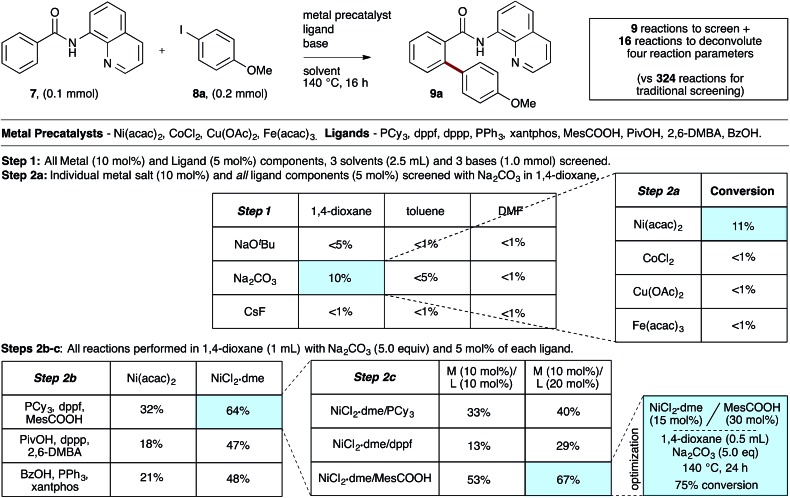
Combinatorial discovery and deconvolution of *in situ*-generated catalysts for C–H monoarylation of unsubstituted benzamide **7**. Conversion determined by ^1^H NMR of the crude mixtures in CDCl_3_. 2,6-DMBA = 2,6-dimethoxybenzoic acid.

To evaluate the catalyst discovery strategy in a more complex system, we elected to explore transition metal catalyzed C–H activation, a reaction class we hypothesized would be highly applicable to such a combinatorial approach owing to the significant molecular recognition between catalyst and substrate. Bidentate chelation assistance for directed C–H activation has found increasing application in the past decade owing to the desirability of functionalizing ‘inert’ C–H bonds.^16^ Since the seminal report of Daugulis,^17^ the 8-aminoquinoline motif has proven effective as a directing group for C–H activation reactions in combination with Pd, Ru, Cu, Ni, Co and Fe catalyst systems.^18^ 8-Aminoquinoline directed *o*-arylation of benzamides with aryl halides was described by Daugulis and coworkers under Pd catalysis^17^ and by Chatani and co-workers under Ru catalysis.^19^ In both cases, a blocking group at the *ortho* or *meta* position of the benzamide is required to avoid undesired products of bisarylation. As these were the sole reports of such a reaction, we felt the search for alternative catalytic systems represented an attractive challenge on which to refine our screening approach. After surveying the literature for metals and ligands employed in directed C–H activation reactions, four metal precatalysts and nine labile ligands were selected as potentially viable co-catalysts for such a transformation (Fig. 2). The coupling of unsubstituted benzamide **7** with 4-iodoanisole (**8a**) was chosen as the target reaction. With a mixture of 10 mol% of each metal precatalyst and 5 mol% of each ligand present in all reactions, three solvents and three bases were screened at 140 °C in nine total reactions (Step 1). Only the combination of Na_2_CO_3_ in 1,4-dioxane gave moderate (10%) conversion to the desired *o*-arylation product **9a**. Thus, a lead result was identified in a four-dimensional screen in just nine reactions. Deconvolution of the metal salts revealed that Ni(acac)_2_ was the active catalyst precursor, whilst Fe(acac)_3_, CoCl_2_ and Cu(OAc)_2_ all exhibited no catalytic activity (Step 2a). Maintaining Na_2_CO_3_ as base and 1,4-dioxane as solvent, the nine ligands were arbitrarily divided into groups of three, and evaluated in combination with Ni(acac)_2_ and NiCl_2_·dme. A significant enhancement of reaction efficiency was observed in the reactions employing PCy_3_, MesCOOH and dppf as ligand (Step 2b). Continued deconvolution established NiCl_2_·dme and MesCOOH as an efficient combination (Step 2c, 67% conversion). Further tweaking of the reaction conditions enabled the conversion to be increased to 75%. Surprisingly, the ratio of **9a** to bisarylation product **10a** was >20 : 1 by ^1^H NMR. Application of these reaction conditions with various aryl iodides allowed for the desired *o*-arylation products to be isolated in 59–76% yields, all with >20 : 1 selectivity for monoarylation (Table 1).

**Table 1:** Reaction scope of monoarylation^*a*^

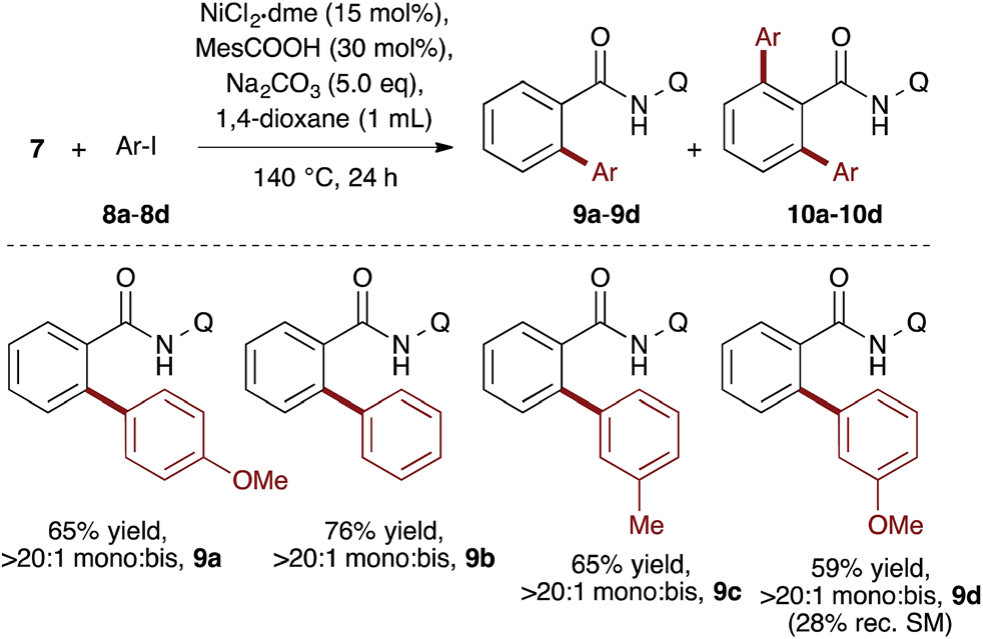

^*a*^Isolated yield after column chromatography. Q = 8-quinoline.

Whilst completing the deconvolution of this reaction system, Chatani and coworkers disclosed a closely related Ni-catalyzed *ortho*-arylation system.^20,21^ Their system proceeds under Ni(OTf)_2_ catalyzed ligand-free conditions at 160 °C and, like existing Pd^17^ and Ru^19^ catalyzed methods, is typically not useful for unsubstituted phenyl benzamides due to the significant formation of bisarylation adducts. Indeed, reaction of **7** with **8a** under Chatani's conditions gave complete conversion to a 3 : 1 mixture of mono- and bisarylation products in our hands (eqn (1)). In contrast, the system described herein exhibits a significant ligand effect,^22^ proceeds at 140 °C and furnishes >20 : 1 selectivity for monoarylation products **9a–9d**. Four parallel screens requiring only 25 total reactions identified a selective set of conditions for directed monoarylation of **7**. Using an inexpensive Ni(ii) salt and 2,4,6-trimethylbenzoic acid, hitherto unreported biaryl products have been accessed in synthetically useful yields.1
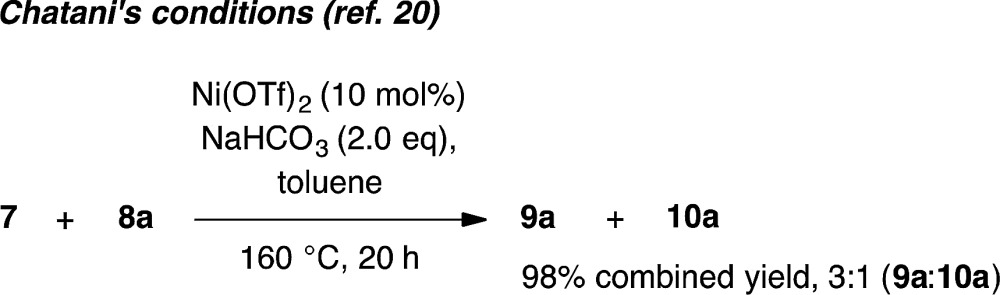



## Conclusion

In conclusion, we have described an approach for catalyst discovery using complex mixtures of potential catalyst components. Our “proof of concept” attempts to apply this strategy have uncovered a mild and novel boron catalyst for the dehydrative Friedel–Crafts reaction and new conditions for Ni-catalyzed directed C–H arylation of benzamide that are highly selective for monoarylation in the absence of blocking groups. Initial hits for both cases were uncovered in 14 reactions or less and required a small number of additional deconvolution reactions to identify key catalyst components. The number of steps in the deconvolution process can be tailored to the preference of the user by adjusting the number of reactions used per deconvolution step. Though the approach's generality and its ability to handle larger sets of catalyst components remains to be seen, screening approaches employing rationally chosen complex mixtures should be useful for chemists lacking the resources for high throughput experimentation, particularly for reactions involving significant molecular recognition between substrate and catalyst. We anticipate this approach will also increase the chances of uncovering unexpected cooperative effects between catalytic components that would not otherwise be assayed in the same reaction vessel.
