# Supplementary material for: Identifying lead hits in catalyst discovery by screening and deconvoluting complex mixtures of catalyst components
Source: Chem Sci. 2015 Feb 16;6(4):2501–5. doi: 10.1039/c5sc00268k (PMC5647744; doi:10.1039/c5sc00268k)

Electronic Supplementary Material (ESI) for Chemical Science.
This journal is © The Royal Society of Chemistry 2015

Identifying Lead Hits in Catalyst Discovery by Screening and Deconvoluting Complex Mixtures of Catalyst Components

Eléna Wolf, Edward Richmond and Joseph Moran*

Institut de Science et d'Ingénierie Supramoléculaires (ISIS), Université de Strasbourg
8 allée Gaspard Monge BP 70028, F-67000 Strasbourg Cedex, France

moran@unistra.fr

Supporting Information

Table of Contents

General Information S1
Materials and Methods S2
Part 1 – Dehydrative Friedel-Crafts Reaction S3
General Procedure for Step 1 S2
General Procedure for Step 2 S3
Linear Optimization S8
Spectral Data for Compound **6** S11
Part 2 – Directed Benzamide C-H Activation/Arylation S12
General Procedure for Step 1 S12
General Procedure for Step 1 S12
Data for Benzamide products **9a-9d** S13
Spectral Data for Novel Compounds S16

General Information. All reactions were performed in 10 mL sealed tubes under an air atmosphere. Purification of reaction products was carried out by column chromatography using Merck silica gel (40-63 μm). Analytical thin layer chromatography (TLC) was performed on aluminum sheets pre-coated with silica gel 60 F254 (E. Merck), cut to size. Visualization was accomplished with UV light followed by dipping in a potassium permanganate and/or Seebach's staining solutions and heating. Elevated temperatures were achieved by way of a stirrer-hotplate, heating block and thermocouple. Solids weighing less than 4 mg were weighed on a semi-micro balance with a readability of 0.01 mg. Melting points were obtained on a Büchi Melting Point B-450 apparatus, IR spectra were recorded on a Shimadzu ATR machine and High Resolution Mass Spectra were obtained on an Agilent Technologies LCMS (ESI) system. ^{1}H NMR spectra were recorded on a Bruker Avance400 (400 MHz) spectrometer at ambient temperature unless otherwise noted and are reported in ppm using solvent as the internal standard ($CDCl_3$ at 7.26 ppm). Data are reported as: multiplicity (ap = apparent, br = broad, s = singlet, d = doublet, t = triplet, q = quartet, m = multiplet), integration and coupling constant(s) in Hz. ^{13}C NMR spectra were recorded on a Bruker Avance400 (100 MHz) spectrometer. Chemical shifts are reported in ppm from

tetramethylsilane, with the residual solvent resonance employed as the internal standard ($CDCl_3$ at 77.0 ppm). ^{19}F NMR spectra were recorded on a Bruker Avance400 (376 MHz) spectrometer. ^{11}B NMR spectra were recorded on a Bruker Avance400 (128 MHz) spectrometer.

Materials and Methods. Unless otherwise noted, all commercial materials were purchased from *Sigma-Aldrich* and used without further purification. 8-Aminoquinoline was purchased from *Combi-Blocks*. 1,4-Dioxane refers to >99.5 (GC grade) stored over molecular sieves (product number 42510-250mL) also purchased from *Sigma-Aldrich*. (*E*)-6-Phenylhex-5-ene-1,4-diol was prepared following a literature procedure.[1] Boronic acids and ligands were used without any special precaution to exclude moisture or air.

[1] M. B. Hay, A.R. Hardin, J. P. Wolfe, *J. Org. Chem.* **2005**, *70*, 3099–3107.

Part 1 – *In Situ* Generated Boron Catalysts from Boronic Acids and Bidentate *O*-Ligands

General procedure for Step 1 (12 boronic acids, 12 *O*-ligands, 14 solvents, 2016 possible combinations)

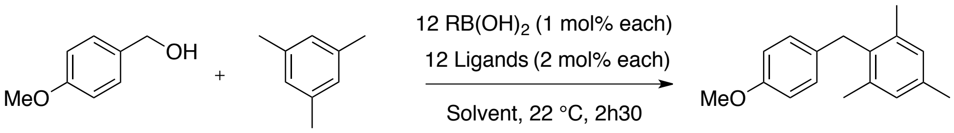

Reactions were carried out in four different solvents simultaneously according to the following procedure using the quantities of boronic acids and ligands described in the table below. Boronic acids **1a**-**1l** (0.010 mmol, 1.0 mol%) and ligands **2a**-**3e** (0.020 mmol, 2.0 mol%) were dissolved in solvent (2.5 mL). Mesitylene (418 µL, 360 mg, 3.00 mmol, 3.00 equiv) and *p*-methoxybenzyl alcohol (124 µL, 138 mg, 1.00 mmol, 1.00 equiv) were added, in that order, by syringe. The reactions were stirred at 22 °C and all were monitored by TLC (Petroleum ether/EtOAc 4:1). After completion of the fastest reaction (2.5 h), all reactions were quenched by dilution with CH_2Cl_2, filtered through a short pad of silica and concentrated under reduced pressure. DMSO (71 µL, 78 mg, 1.0 mmol, 1.0 equiv) was added as an internal standard and the mixture was taken up in $CDCl_3$. ^{1}H NMR of these solutions were recorded and the % yield calculated based on the ratio of the DMSO resonance (δ 2.61 ppm, 6H) to the resonance corresponding to the benzylic methylene of compound **6** (δ 4.01, 2H). In the case of the fastest reaction (in $MeNO_2$), compound **6** was isolated as a white solid after flash column chromatography (CH_2Cl_2/Petroleum ether 10:1; SiO_2). R_f = 0.42 (Petroleum ether/EtOAc 20:1). Yields are described below for each case.

1-(4-Methoxybenzyl)-2,4,6-trimethylbenzene (**6**). **^{1}H NMR** (400 MHz, $CDCl_3$): δ = 7.01–6.93 (m, 4H), 6.86–6.81 (m, 2H), 4.01 (s, 2H), 3.80 (s, 3H), 2.35 (s, 3H), 2.26 (s, 6H); **^{13}C NMR** (100 MHz, $CDCl_3$): δ = 157.8, 137.1, 135.7, 134.3, 132.2, 129.0, 128.9, 113.9, 55.3, 33.9, 21.0, 20.2. The analytical data are in accordance with those reported in the literature.[2]

[2] M. Hofmann, N. Hampel, T. Kanzian, H. Mayr, *Angew. Chem. Int. Ed.* **2004**, *43*, 5402–5405.

*Screening of boronic acids **1a-1l** (1 mol%) – ligands **2a-3e** (2 mol%)*

1a $C_6F_5B(OH)_2$: 2.1 mg
1b 2,3,4-F_3-$C_6HB(OH)_2$: 1.8 mg
1c 4-F-$C_6H_4B(OH)_2$: 1.4 mg
1d 3,4-Cl_2-$C_6H_3B(OH)_2$: 1.9 mg
1e 4-OMe-$C_6H_4B(OH)_2$: 1.5 mg
1f 4-CO_2H-$C_6H_4B(OH)_2$: 1.7 mg
1g 2,3,4,5- F_4-$C_6HB(OH)_2$: 1.9 mg
1h 3,4-F_2-$C_6H_2B(OH)_2$: 1.6 mg
1i 4-Cl-$C_6H_4B(OH)_2$: 1.6 mg
1j $C_6H_5B(OH)_2$: 1.2 mg
1k 2-NO_2-$C_6H_4B(OH)_2$: 1.7 mg
1l $B(OH)_3$: 0.6 mg

2a Oxalic acid dihydrate : 2.5 mg
2b Tartaric acid : 3.0 mg
2c Glycolic acid : 1.5 mg
2d Glyoxylic acid : 1.8 mg
2e Pinacol : 2.4 mg
2f Catechol : 2.2 mg
2g Tartaric acid dimethylester : 3.6 mg
3a 3-Hydroxypropanoic acid : 1.8 mg
3b Salicylic acid : 2.8 mg
3c Malonic acid : 2.1 mg
3d Succinic acid : 2.4 mg
3e Phtalic acid : 3.3 mg

→ $MeNO_2$: 77% yield (isolated)
→ MeCN: 26% yield (NMR)
→ DCM: 3% yield (NMR)
→ DCE: 3% yield (NMR)
→ Toluene: <1% yield (NMR)
→ Benzene: <1% yield (NMR)
→ DMF: <1% yield (NMR)
→ Acetone: <1% yield (NMR)
→ Et_2O: <1% yield (NMR)
→ THF: <1% yield (NMR)
→ EtOAc: <1% yield (NMR)
→ 1,4-Dioxane: <1% yield (NMR)
→ iPrOH: <1% yield (NMR)
→ H_2O: <1% yield (NMR)

General Procedure for Step 2

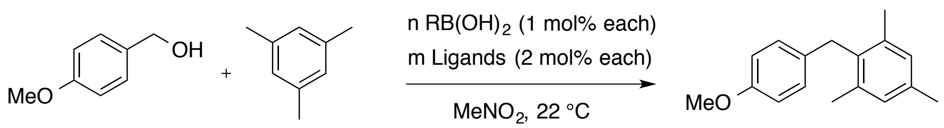

For each deconvolution step, reactions were carried out simultaneously according to the following procedure using the boronic acids and ligands described in the tables below. Boronic acids (0.01 mmol, 1.0 mol%) and ligands (0.02 mmol, 2.0 mol%) were dissolved in $MeNO_2$ (2.5 mL). Mesitylene (418 μL, 360 mg, 3.00 mmol, 3.00 equiv), followed by *p*-methoxybenzyl alcohol (124 μL, 138 mg, 1.00 mmol, 1.00 equiv) were added by syringe. The reactions were stirred at 22 °C and all were monitored by TLC (Petroleum ether/EtOAc 4:1). After completion of the fastest reaction for each step, all reactions were quenched by dilution with CH_2Cl_2, filtered through a pad of silica and concentrated under reduced pressure. DMSO (71 μL, 78 mg, 1.0 mmol, 1.0 equiv) was added as an internal standard and the mixture was taken up in $CDCl_3$. ^1H NMR of these solutions were recorded and the % yield calculated based on the ratio of the DMSO resonance (δ 2.61 ppm, 6H) to the resonance corresponding to the benzylic methylene of compound **6** (δ 4.01, 2H). Yields are described below for each case.

Step I - 4 reactions, reaction time: 2.5 h

*Screening of boronic acids **1a**-**1f** (1 mol%) – ligands **2a**-**2g** (2 mol%)*

1a $C_6F_5B(OH)_2$: 2.1 mg
1b 2,3,4-F_3-$C_6HB(OH)_2$: 1.8 mg
1c 4-F-$C_6H_4B(OH)_2$: 1.4 mg
1d 3,4-Cl_2-$C_6H_3B(OH)_2$: 1.9 mg
1e 4-OMe-$C_6H_4B(OH)_2$: 1.5 mg
1f 4-CO_2H-$C_6H_4B(OH)_2$: 1.7 mg

2a Oxalic acid dihydrate : 2.5 mg
2b Tartaric acid : 3.0 mg
2c Glycolic acid : 1.5 mg
2d Glyoxylic acid : 1.8 mg
2e Pinacol : 2.4 mg
2f Catechol : 2.2 mg
2g Tartaric acid dimethylester : 3.6 mg

→ 90 % yield

*Screening of boronic acids **1g**-**1l** (1 mol%) – ligands **2a**-**2g** (2 mol%)*

1g 2,3,4,5- F_4-$C_6HB(OH)_2$: 1.9 mg
1h 3,4-F_2-$C_6H_2B(OH)_2$: 1.6 mg
1i 4-Cl-$C_6H_4B(OH)_2$: 1.6 mg
1j $C_6H_5B(OH)_2$: 1.2 mg
1k 2-NO_2-$C_6H_4B(OH)_2$: 1.7 mg
1l $B(OH)_3$: 0.6 mg

2a Oxalic acid dihydrate : 2.5 mg
2b Tartaric acid : 3.0 mg
2c Glycolic acid : 1.5 mg
2d Glyoxylic acid : 1.8 mg
2e Pinacol : 2.4 mg
2f Catechol : 2.2 mg
2g Tartaric acid dimethylester : 3.6 mg

→ 63 % yield

*Screening of boronic acids **1a**-**1f** (1 mol%) – ligands **3a**-**3e** (2 mol%)*

1a $C_6F_5B(OH)_2$: 2.1 mg
1b 2,3,4-F_3-$C_6HB(OH)_2$: 1.8 mg
1c 4-F-$C_6H_4B(OH)_2$: 1.4 mg
1d 3,4-Cl_2-$C_6H_3B(OH)_2$: 1.9 mg
1e 4-OMe-$C_6H_4B(OH)_2$: 1.5 mg
1f 4-CO_2H-$C_6H_4B(OH)_2$: 1.7 mg

3a 3-Hydroxypropanoic acid : 1.8 mg
3b Salicylic acid : 2.8 mg
3c Malonic acid : 2.1 mg
3d Succinic acid : 2.4 mg
3e Phtalic acid : 3.3 mg

→ 7 % yield

*Screening of boronic acids **1g**-**1l** (1 mol%) – ligands **3a**-**3e** (2 mol%)*

1g 2,3,4,5- F_4-$C_6HB(OH)_2$: 1.9 mg
1h 3,4-F_2-$C_6H_2B(OH)_2$: 1.6 mg
1i 4-Cl-$C_6H_4B(OH)_2$: 1.6 mg
1j $C_6H_5B(OH)_2$: 1.2 mg
1k 2-NO_2-$C_6H_4B(OH)_2$: 1.7 mg
1l $B(OH)_3$: 0.6 mg

3a 3-Hydroxypropanoic acid : 1.8 mg
3b Salicylic acid : 2.8 mg
3c Malonic acid : 2.1 mg
3d Succinic acid : 2.4 mg
3e Phtalic acid : 3.3 mg

→ 5 % yield

Step I<br>2h30	1a-1f<br>$RB(OH)_2$	1g-1l<br>$RB(OH)_2$
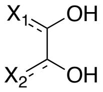<br>**2a-2g**	**90 %**	63%
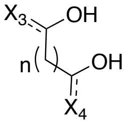<br>**3a-3e**	7%	5%

Step II - 9 reactions, reaction time: 1 h

*Screening of boronic acids **1a** and **1b** (1 mol%) – ligands **2a** and **2b** (2 mol%)*

1a $C_6F_5B(OH)_2$: 2.1 mg
1b 2,3,4-F_3-$C_6HB(OH)_2$: 1.8 mg

2a Oxalic acid dihydrate : 2.5 mg
2b Tartaric acid : 3.0 mg

→ 91 % yield

*Screening of boronic acids **1c** and **1d** (1 mol%) – ligands **2a** and **2b** (2 mol%)*

1c 4-F-$C_6H_4B(OH)_2$: 1.4 mg
1d 3,4-Cl_2-$C_6H_3B(OH)_2$: 1.9 mg

2a Oxalic acid dihydrate : 2.5 mg
2b Tartaric acid : 3.0 mg

→ 33 % yield

*Screening of boronic acids **1e** and **1f** (1 mol%) – ligands **2a** and **2b** (2 mol%)*

1e 4-OMe-$C_6H_4B(OH)_2$: 1.5 mg
1f 4-CO_2H-$C_6H_4B(OH)_2$: 1.7 mg

2a Oxalic acid dihydrate : 2.5 mg
2b Tartaric acid : 3.0 mg

→ 29 % yield

*Screening of boronic acids **1a** and **1b** (1 mol%) – ligands **2c** and **2d** (2 mol%)*

1a $C_6F_5B(OH)_2$: 2.1 mg
1b 2,3,4-F_3-$C_6HB(OH)_2$: 1.8 mg

2c Glycolic acid : 1.5 mg
2d Glyoxylic acid : 1.8 mg

→ 8 % yield

*Screening of boronic acids **1c** and **1d** (1 mol%) – ligands **2c** and **2d** (2 mol%)*

1c 4-F-$C_6H_4B(OH)_2$: 1.4 mg
1d 3,4-Cl_2-$C_6H_3B(OH)_2$: 1.9 mg

2c Glycolic acid : 1.5 mg
2d Glyoxylic acid : 1.8 mg

→ 2 % yield

*Screening of boronic acids **1e** and **1f** (1 mol%) – ligands **2c** and **2d** (2 mol%)*

1e 4-OMe-$C_6H_4B(OH)_2$: 1.5 mg
1f 4-CO_2H-$C_6H_4B(OH)_2$: 1.7 mg

2c Glycolic acid : 1.5 mg
2d Glyoxylic acid : 1.8 mg

→ 3 % yield

*Screening of boronic acids **1a** and **1b** (1 mol%) – ligands **2e, 2f** and **2g** (2 mol%)*

1a $C_6F_5B(OH)_2$: 2.1 mg
1b 2,3,4-F_3-$C_6HB(OH)_2$: 1.8 mg

2e Pinacol : 2.4 mg
2f Catechol : 2.2 mg
2g Tartaric acid dimethylester : 3.6 mg

→ <1 % yield

*Screening of boronic acids **1c** and **1d** (1 mol%) – ligands **2e, 2f** and **2g** (2 mol%)*

1c 4-F-$C_6H_4B(OH)_2$: 1.4 mg
1d 3,4-Cl_2-$C_6H_3B(OH)_2$: 1.9 mg

2e Pinacol : 2.4 mg
2f Catechol : 2.2 mg
2g Tartaric acid dimethylester : 3.6 mg

→ <1 % yield

*Screening of boronic acids **1e** and **1f** (1 mol%) – ligands **2e, 2f** and **2g** (2 mol%)*

1e 4-OMe-$C_6H_4B(OH)_2$: 1.5 mg
1f 4-CO_2H-$C_6H_4B(OH)_2$: 1.7 mg

2e Pinacol : 2.4 mg
2f Catechol : 2.2 mg
2g Tartaric acid dimethylester : 3.6 mg

→ <1 % yield

**Step II** 1h	**1a, 1b** $RB(OH)_2$	**1c, 1d** $RB(OH)_2$	**1e, 1f** $RB(OH)_2$
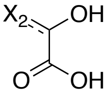 **2a, 2b**	**91 %**	33%	29%
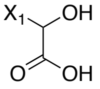 **2c, 2d**	8%	2%	3%
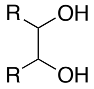 **2e, 2f, 2g**	<1%	<1%	<1%

Step III - 4 reactions, reaction time: 15 min

*Screening of boronic acids **1a** and **1b** (1 mol%) – ligands **2a** and **2b** (2 mol%)*

1a $C_6F_5B(OH)_2$: 2.1 mg
→ 95 % yield

2a Oxalic acid dihydrate : 2.5 mg

1a $C_6F_5B(OH)_2$: 2.1 mg
→ 19 % yield

2b Tartaric acid : 3.0 mg

1b 4-F-$C_6H_4B(OH)_2$: 1.4 mg
→ 16 % yield

2a Oxalic acid dihydrate : 2.5 mg

1b 4-F-$C_6H_4B(OH)_2$: 1.4 mg
→ <1 % yield

2b Tartaric acid : 3.0 mg

**Step III** 15 min	**1a**	**1b**
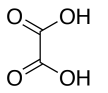 **2a**	**95 %**	16%
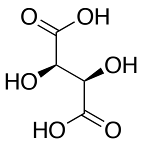 **2b**	19%	<1%

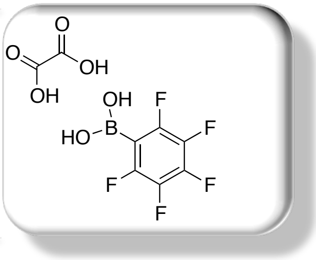

Linear optimization

a) *Representative procedure for ligand optimization*

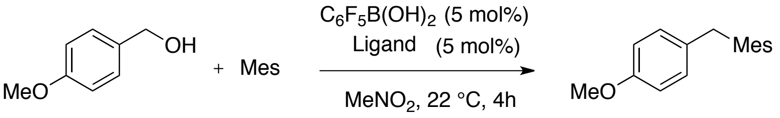

Mesitylene (209 µL, 180 mg, 1.50 mmol, 3.00 equiv) and *p*-methoxybenzyl alcohol (62 µL, 69 mg, 0.50 mmol, 1.0 equiv) were added by syringe into $MeNO_2$ (2.5 mL). Pentafluorophenylboronic acid **1a** (0.025 mmol, 5.0 mol%) and ligand (0.025 mmol, 5.0 mol%) were added to the solution. The reaction mixture was stirred for 4 h at 22 °C unless otherwise noted. Yields are described below for each case.

Control experiment : Mesitylene (209 µL, 180 mg, 1.5 mmol, 3.00 equiv) and *p*-methoxybenzyl alcohol (62 µL, 69 mg, 0.50 mmol, 1.0 equiv) were added by syringe into $MeNO_2$ (2.5 mL). Ligand **2a-3e** (0.050 mmol, 10 mol%) was added to the solution. The reaction mixture was stirred for 4 h at 22 °C. Yields are described below for each case.

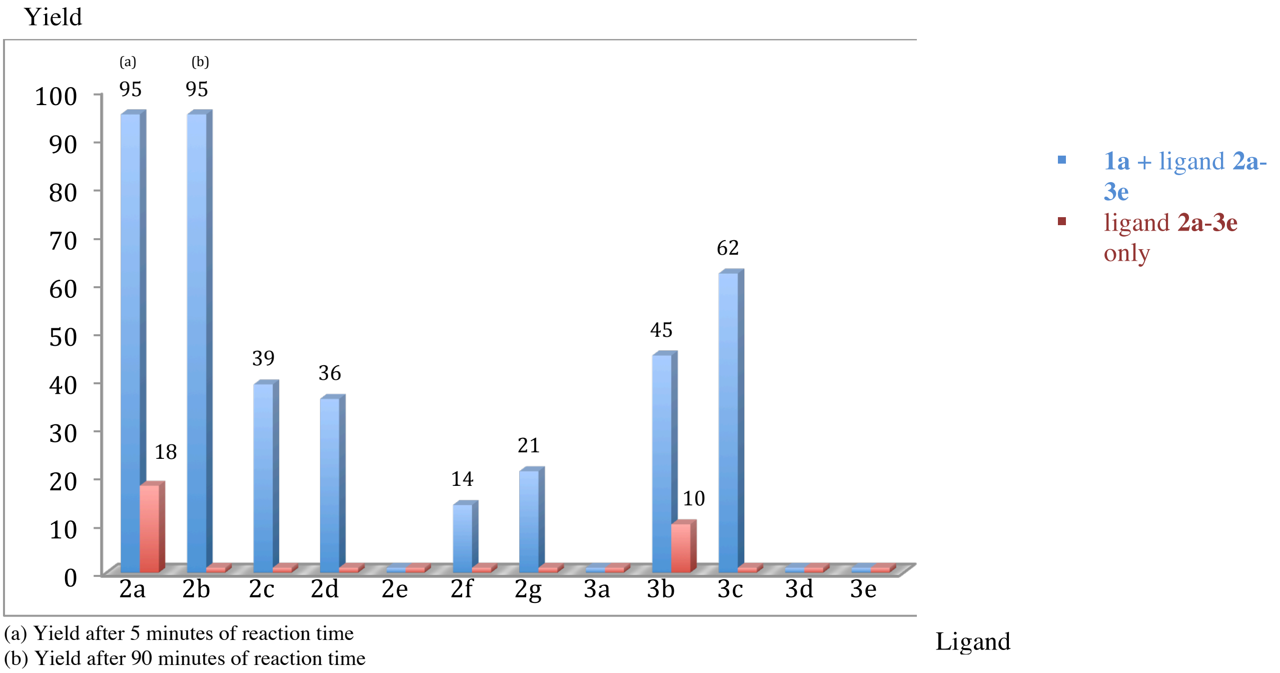

(a) Yield after 5 minutes of reaction time
(b) Yield after 90 minutes of reaction time

b) Representative procedure for boronic acid optimization

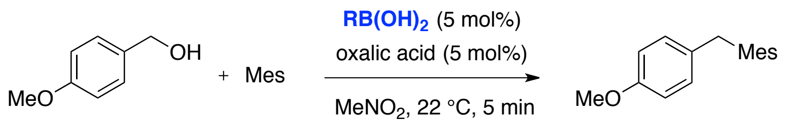

Mesitylene (209 µL, 180 mg, 1.50 mmol, 3.00 equiv) and *p*-methoxybenzyl alcohol (62 µL, 69 mg, 0.50 mmol, 1.0 equiv) were added by syringe into $MeNO_2$ (2.5 mL). Boronic acid **1a-1l** (0.025 mmol, 5.0 mol%) and oxalic acid (0.025 mmol, 5.0 mol%) were added to the solution. The reaction mixture was stirred for 5 minutes at 22 °C. Yields are described below for each case.

Control experiment : Mesitylene (209 µL, 180 mg, 1.50 mmol, 3.00 equiv) and *p*-methoxybenzyl alcohol (62 µL, 69 mg, 0.50 mmol, 1.0 equiv) were added by syringe into $MeNO_2$ (2.5 mL). Boronic acid **1a-1l** (0.050 mmol, 10 mol%) was added to the solution. The reaction mixture was stirred for 4 h at 22 °C. Yields are described below for each case.

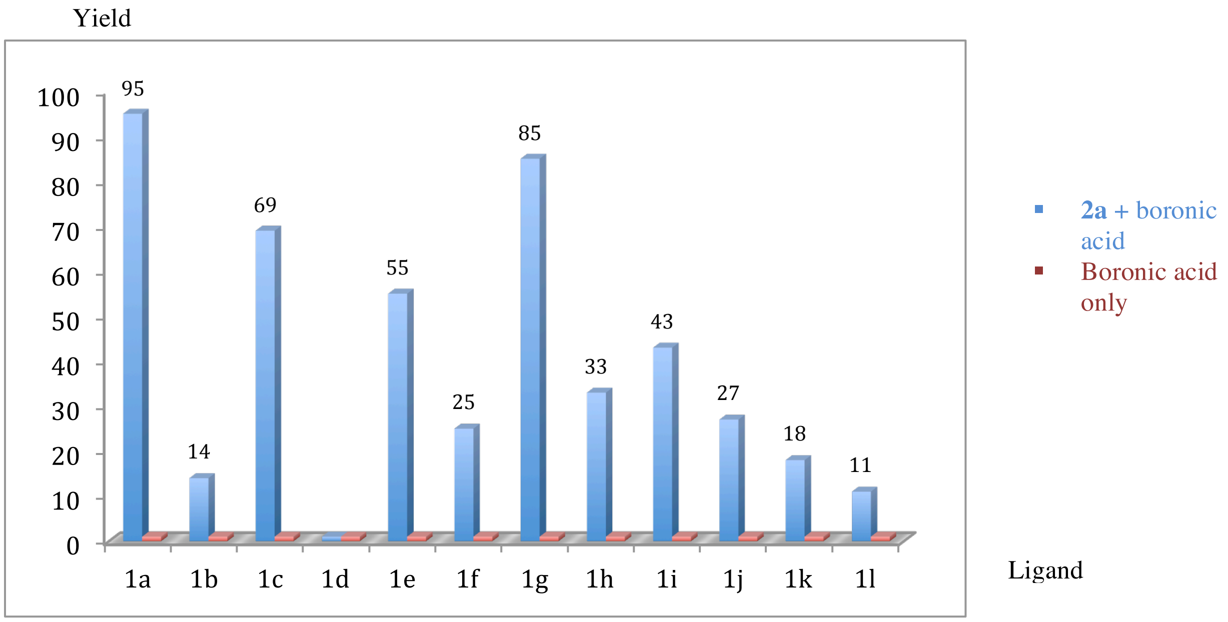

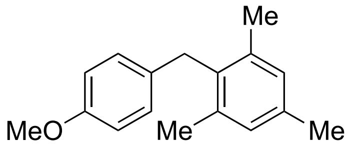
Me
MeO
Me
Me

6

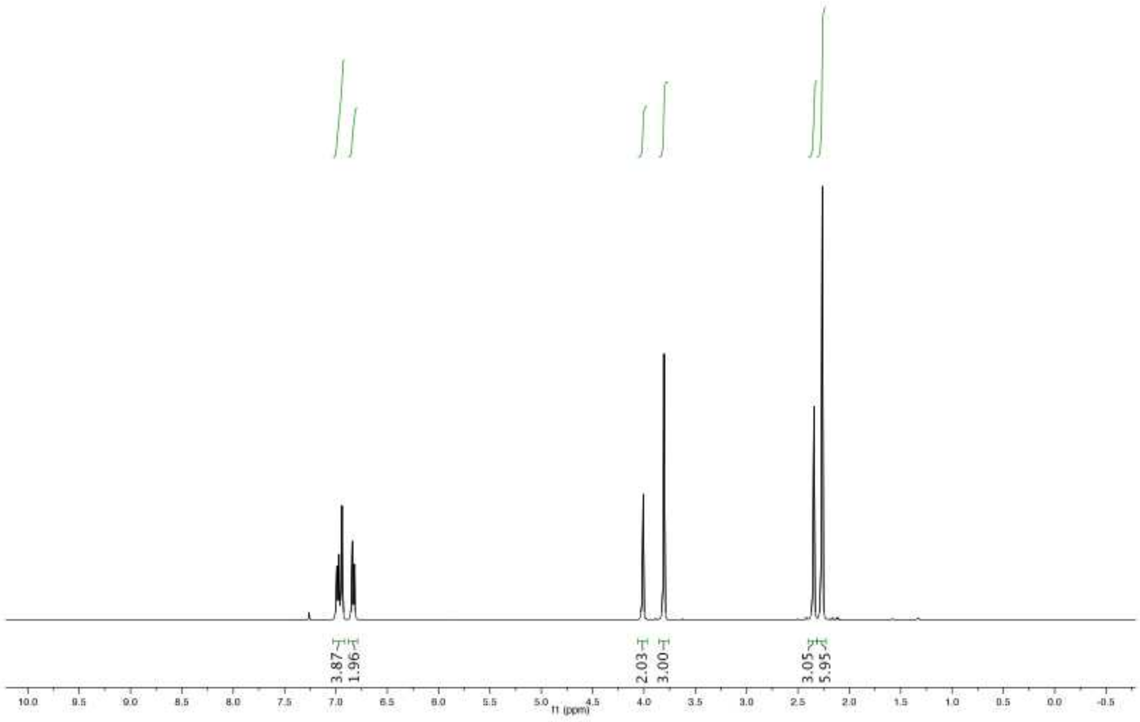
3.87
1.96
2.03
3.00
3.05
5.95
f1 (ppm)

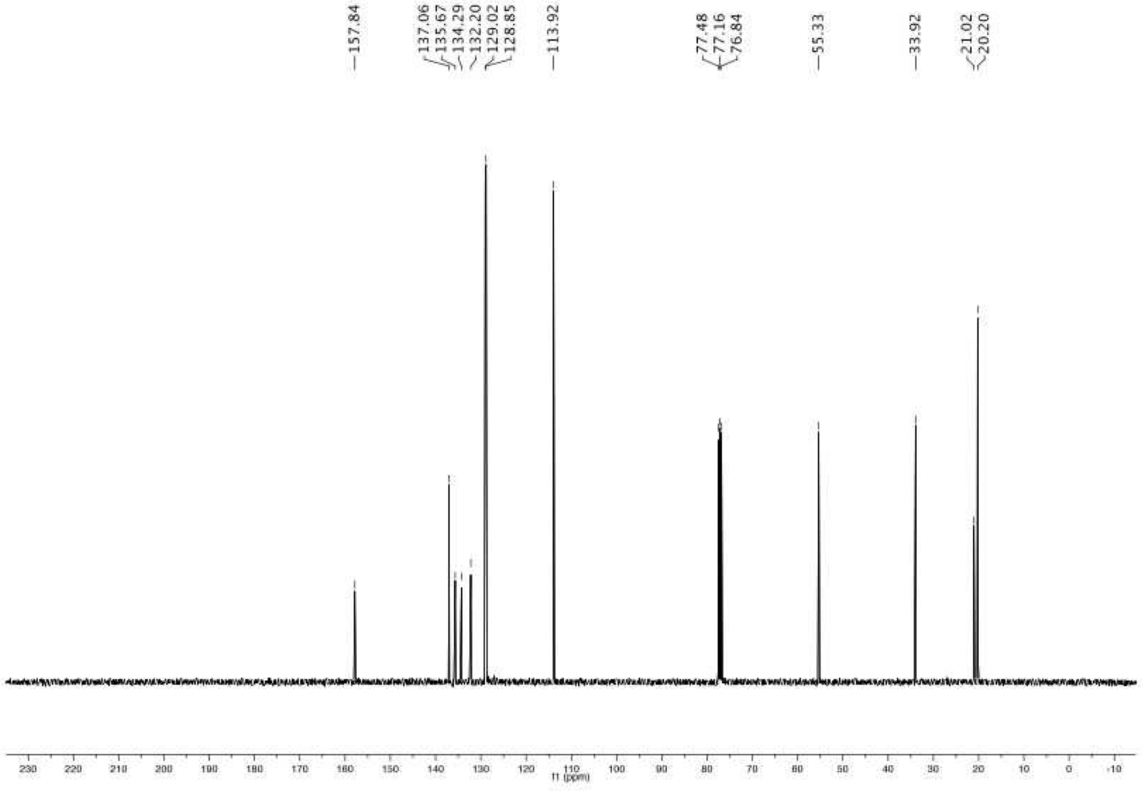
157.84
137.06
135.67
134.29
132.20
129.02
128.85
113.92
77.48
77.16
76.84
55.33
33.92
21.02
20.20
f1 (ppm)

Part 2 – Directed Benzamide C-H Activation/Arylation

Step 1 Procedure

To an oven dried 10 mL screw-top vial equipped with a magnetic stirrer bar was added benzamide **7** (0.1 mmol), 4-iodoanisole (0.2 mmol), Na_2CO_3 (0.5 mmol), metal salts (10 mol%) and ligands (5 mol %). 1,4-dioxane (2.5 mL) was then introduced by syringe, the vial sealed and stirred for 10 min at room temperature to allow for complete dissolution. The vial was then transferred to a heating block at 140 °C and stirred rapidly (1200 rpm) at this temperature for 16 h. The reaction progress was monitored by TLC analysis (20% EtOAc in petrol). After allowing to cool, the crude reaction mixture was filtered through a celite plug, the filter cake washed with CH_2Cl_2 and the crude reaction mixture concentrated *in vacuo*. By ^1H NMR analysis, the approximate conversion of each reaction could be calculated by the relative integrations of the characteristic amide N*H* signals of the starting material and product.

Representative Procedure

To an oven dried 10 mL screw-top vial equipped with a magnetic stirrer bar was added benzamide **7** (0.1 mmol, 0.025 g), 4-iodoanisole (0.2 mmol, 0.044 g), Na_2CO_3 (0.5 mmol, 0.053 g), $Ni(acac)_2$ (10 mol%, 2.6 mg), $Fe(acac)_3$ (10 mol%, 3.5 mg), $CoCl_2$ (10 mol%, 1.3 mg), $Cu(OAc)_2$ (10 mol%, 1.8 mg), benzoic acid (5 mol%, 0.6 mg), PivOH (5 mol%, 0.5 mg), 2,6-dimethoxybenzoic acid (5 mol%, 0.9 mg), 2,4,6-trimethylbenzoic acid (5 mol%, 0.8 mg), PPh_3 (5 mol%, 1.3 mg), PCy_3 (5 mol%, 1.4 mg), xantphos (5 mol%, 2.9 mg), dppf (5 mol%, 2.7 mg), and dppp (5 mol%, 2.2 mg). 1,4-dioxane (2.5 mL) was then introduced by syringe, the vial sealed and stirred for 10 min at room temperature to allow for complete dissolution. The vial was then transferred to a heating block at 140 °C and stirred rapidly (1200 rpm) at this temperature for 16 h. The reaction progress was monitored by TLC analysis (20% EtOAc in petrol). After allowing to cool, the crude reaction mixture was filtered through a celite plug, the filter cake washed with CH_2Cl_2 and the crude reaction mixture concentrated *in vacuo*. The conversion of the reaction was calculated as approximately 10% from the crude ^1H NMR spectrum.

Step 2 Procedure

To an oven dried 10 mL screw-top vial equipped with a magnetic stirrer bar was added benzamide **7** (0.1 mmol), 4-iodoanisole (0.2 mmol), Na_2CO_3 (0.5 mmol), metal salts (10 mol%) and ligands (desired mol %). 1,4-dioxane (1.0 mL) was then introduced by syringe, the vial sealed and stirred for 10 min at room temperature to allow for complete dissolution. The vial was then transferred to a heating block at 140 °C and stirred rapidly (1200 rpm) at this temperature for 16 h. The reaction progress was monitored by TLC analysis (20% EtOAc in petrol). After allowing to cool, the crude reaction mixture was filtered through a celite plug, the filter cake washed with CH_2Cl_2 and the crude reaction mixture concentrated *in vacuo*. By ^1H NMR analysis, the approximate conversion of each reaction could be calculated by the relative integrations of the characteristic amide N*H* signals of the starting material and product.

Representative Procedure

To an oven dried 10 mL screw-top vial equipped with a magnetic stirrer bar was added benzamide **7** (0.1 mmol, 0.025 g), 4-iodoanisole (0.2 mmol, 0.044 g), Na_2CO_3 (0.5 mmol, 0.053 g), $NiCl_2$•dme (10 mol%, 2.2 mg), 2,4,6-trimethylbenzoic acid (5 mol% 0.8 mg), PCy_3 (5 mol%, 1.4 mg) and dppf (5 mol%, 2.7 mg). 1,4-dioxane (2.5 mL) was then introduced by syringe, the vial sealed and stirred for 10 min at room temperature to allow for complete dissolution. The vial was then transferred to a heating block at 140 °C and stirred rapidly (1200 rpm) at this temperature for 16 h. The reaction progress was monitored by TLC analysis (20% EtOAc in petrol). After allowing to cool, the crude reaction mixture was filtered through a celite plug, the filter cake washed with CH_2Cl_2 and the crude reaction mixture concentrated *in vacuo*. The conversion of the reaction was calculated as approximately 64% from the crude ^{1}H NMR spectrum.

Optimized Reaction Conditions – General Procedure A

To an oven dried 10 mL screw-top vial equipped with a magnetic stirrer bar was added benzamide **7** (0.2 mmol), aryliodide (**8a-d**) (0.4 mmol), Na_2CO_3 (1.0 mmol), $NiCl_2$•dme (15 mol%) and MesCOOH (30 mol %). 1,4-dioxane (1.0 mL) was then introduced by syringe, the vial sealed and stirred for 10 min at room temperature to allow for complete dissolution. The vial was then transferred to a heating block at 140 °C and stirred rapidly (1200 rpm) at this temperature for 24 h. The reaction progress was monitored by TLC analysis (20% EtOAc in petrol). After allowing to cool, the crude reaction mixture was filtered through a celite plug, the filter cake washed with CH_2Cl_2 and the crude reaction mixture concentrated *in vacuo* directly onto silica gel. The arylated benzamides (**9a-d**) were then purified by flash column chromatography over silica with the eluent systems stated.

Products

***N*-(Quinolin-8-yl)benzamide 7**

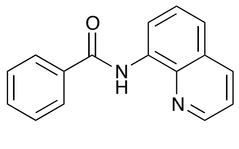

To a stirred solution of 8-aminoquinoline (3.00g, 21.0 mmol) in CH_2Cl_2 (20 mL) was added DMAP (80.0 mg, 0.65 mmol) followed by Et_3N (3.30 mL, 24.0 mmol). The reaction mixture was cooled to 0 °C and benzoyl chloride (2.30 mL, 20 mmol) was added dropwise. The reaction mixture was stirred for 16 h allowing to warm to rt. After such time, the reaction was quenched with sat. aq. $NaHCO_3$ (10 mL) and stirred rapidly for 10 min. The organic layer was separated, diluted with CH_2Cl_2 (20 mL) and washed with 1M HCl (20 mL) followed by brine (20 mL). The organic layers were dried over Na_2SO_4, filtered and concentrated *in vacuo* prior to purification by flash column chromatography over silica (20% EtOAc in petrol) to yield benzamide **7** as a white solid (3.25 g, 65%) with spectral data in accordance with the literature.[3]

[3] Y-M. Liang and co-workers, *Org. Lett.* **2009**, *11*, 5726.

^{1}H NMR (400 MHz, $CDCl_3$) δ 10.76 (1H, br s), 8.95 (1H, dd, J = 7.5, 1.5), 8.86 (1H, dd, J = 4.2, 1.7), 8.20 (1H, dd, J = 8.3, 1.7), 8.11-8.08 (2H, m), 7.63-7.54 (5H, m), 7.49 (1H, dd, J = 8.3, 4.2).

4'-Methoxy-*N*-(quinolin-8-yl)-[1,1'-biphenyl]-2-carboxamide 9a

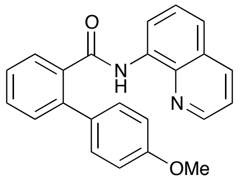

Bis-aryl **9a** was prepared according to general procedure **A** from benzamide **7** and 4-iodoansiole (**8a**). Purification by column chromatography over silica (0-20% EtOAc in petrol) gave **9a** as a white solid (65% yield); mp 113-114 °C; ν_{max} cm^{-1} (ATR) 3337, 1668; ^{1}H NMR (400 MHz, $CDCl_3$) δ 9.81 (1H, br s), 8.82 (1H, dd, J = 7.5, 1.0), 8.54 (1H, dd, J = 4.2, 1.6), 8.09 (1H, dd, J = 8.3, 1.4), 7.90 (1H, dd, J = 7.9, 1.2), 7.57-7.51 (2H, m), 7.48-7.43 (5H, m), 7.36 (1H, dd, J = 8.3, 4.2), 6.82 (2H, d, J = 8.7), 3.66 (3H, s); ^{13}C NMR (125 MHz, $CDCl_3$) δ 168.0, 159.4, 147.7, 139.9, 138.5, 136.0, 136.0, 134.7, 132.4, 130.6, 130.5, 130.2, 129.3, 127.8, 127.3, 127.2, 121.5, 121.4, 116.3, 114.0, 55.2; HRMS (ESI$^+$) $C_{23}H_{18}O_2N_2^+$ found 354.1372 requires 354.1368 (-1.0 ppm).

4,4''-Dimethoxy-*N*-(quinolin-8-yl)-[1,1':3',1''-terphenyl]-2'-carboxamide 10a

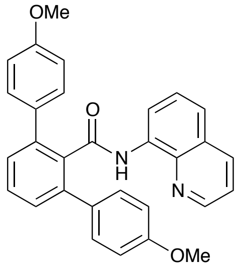

10a was isolated as a byproduct from a reaction according to general procedure **A**. A white solid (trace, <5% yield); mp 189-190 °C; ν_{max} cm^{-1} (ATR) 3321,1672; ^{1}H NMR (400 MHz, $CDCl_3$) δ 9.62 (1H, br s), 8.59-8.55 (2H, m), 8.06 (1H, dd, J = 8.3, 1.4), 7.54-7.40 (9H, m), 7.35 (1H, dd, J = 8.2, 4.2), 6.78 (4H, d, J = 8.7), 3.67 (6H, s); ^{13}C NMR (125 MHz, $CDCl_3$) δ 167.9, 159.0, 147.8, 140.2, 138.4, 136.1, 136.0, 134.4, 132.9, 129.8, 129.2, 129.1, 127.7, 127.2, 121.4, 121.3, 116.5, 113.7, 55.1; HRMS (ESI$^+$) $C_{30}H_{24}O_3N_2^+$ found 460.1799 requires 460.1787 (-2.7 ppm).

***N*-(Quinolin-8-yl)-[1,1'-biphenyl]-2-carboxamide 9b**

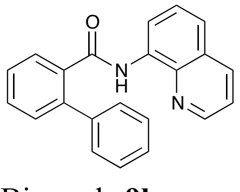

Bis-aryl **9b** was prepared according to general procedure **A** from benzamide **7** and iodobenzene (**8b**). Purification by column chromatography over silica (0-10% EtOAc in

petrol) gave **9b** as a white solid (75% yield); mp 117-118 °C; ν_{max} cm^{-1} (ATR) 3321, 1661; ^{1}H NMR (400 MHz, $CDCl_3$) δ 9.78 (1H, br s), 8.81 (1H, d, J = 7.5), 8.53 (1H, dd, J = 4.1, 1.4), 8.08 (1H, dd, J = 8.3, 1.4), 7.91 (1H, d, J = 7.8), 7.59-7.45 (8H, m), 7.35 (1H, dd, J = 8.3, 4.2), 7.30-7.26 (1H, m), 7.16 (1H, t, J = 7.4); ^{13}C NMR (125 MHz, $CDCl_3$) δ 167.8, 147.8, 140.3, 140.1, 138.4, 136.2, 136.0, 134.6, 130.7, 130.5, 129.2, 129.0, 128.4, 127.7, 127.6, 127.6, 127.3, 121.5, 121.4, 116.3; HRMS (ESI$^+$) $C_{22}H_{16}ON_2^+$ found 248.0951 requires 248.0950 (-0.6 ppm).

3'-Methyl-*N*-(quinolin-8-yl)-[1,1'-biphenyl]-2-carboxamide 9c

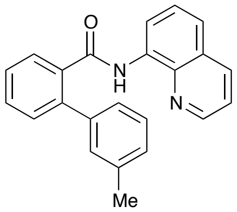

Bis-aryl **9c** was prepared according to general procedure **A** from benzamide **7** and 3-iodotoluene (**8c**). Purification by column chromatography over silica (0-10% EtOAc in petrol) gave **9c** as a white solid (65% yield); mp 92-93 °C; ν_{max} cm^{-1} (ATR) 3331, 1661; ^{1}H NMR (400 MHz, $CDCl_3$) δ 9.77 (1H, br s), 8.81 (1H, d, J = 7.5), 8.54 (1H, dd, J = 4.1, 1.3), 8.08 (1H, dd, J = 8.2, 1.1), 7.90 (1H, d, J = 7.5), 7.58-7.45 (5H, m), 7.37-7.34 (2H, m), 7.29 (1H, d, J = 7.6), 7.13 (1H, t, J = 7.6), 6.96 (1H, d, J = 7.3), 2.25 (3H, s); ^{13}C NMR (125 MHz, $CDCl_3$) δ 167.9, 147.7, 140.5, 140.0, 138.5, 138.0, 136.2, 136.0, 134.7, 130.6, 130.5, 129.7, 129.2, 128.3, 128.2, 127.7, 127.5, 127.3, 126.1, 121.5, 121.4, 116.3, 21.4; HRMS (ESI$^+$) $C_{23}H_{18}O_2N_2^+$ found 248.0950 requires 248.0950 (-0.3 ppm).

3'-Methoxy-*N*-(quinolin-8-yl)-[1,1'-biphenyl]-2-carboxamide 9d

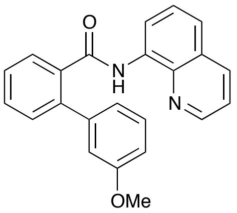

Bis-aryl **9d** was prepared according to general procedure **A** from benzamide **7** and 3-iodotoluene (**8d**). Purification by column chromatography over silica (0-20% EtOAc in petrol) gave **9d** as a white solid (59% yield); mp 111-112 °C; ν_{max} cm^{-1} (ATR) 3323, 1661; ^{1}H NMR (400 MHz, $CDCl_3$) δ 9.79 (1H, br s), 8.82 (1H, d, J = 7.4), 8.54 (1H, dd, J = 4.0, 0.9), 8.08 (1H, d, J = 8.2), 7.91 (1H, d, J = 7.3), 7.58-7.34 (5H, m), 7.36 (1H, dd, J = 8.2, 4.2), 7.16 (1H, t, J = 8.0), 7.09 (2H, br s), 6.70 (1H, d, J = 7.3), 3.70 (3H, s); ^{13}C NMR (125 MHz, CD_3CN) δ 167.5, 159.8, 148.5, 141.5, 139.8, 138.1, 136.3, 136.2, 134.6, 130.6, 130.5, 129.5, 128.7, 127.8, 127.0, 122.0, 121.8, 121.2, 115.5, 114.3, 113.3, 54.9 (*only 22 ^{13}C signals are observed*); HRMS (ESI$^+$) $C_{23}H_{18}O_2N_2^+$ found 354.1374 requires 354.1368 (-1.6 ppm).

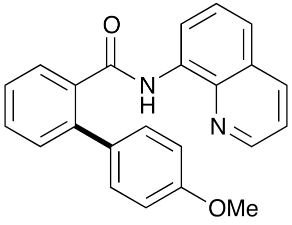

9a

^{13}C, $CDCl_3$, 125 MHz

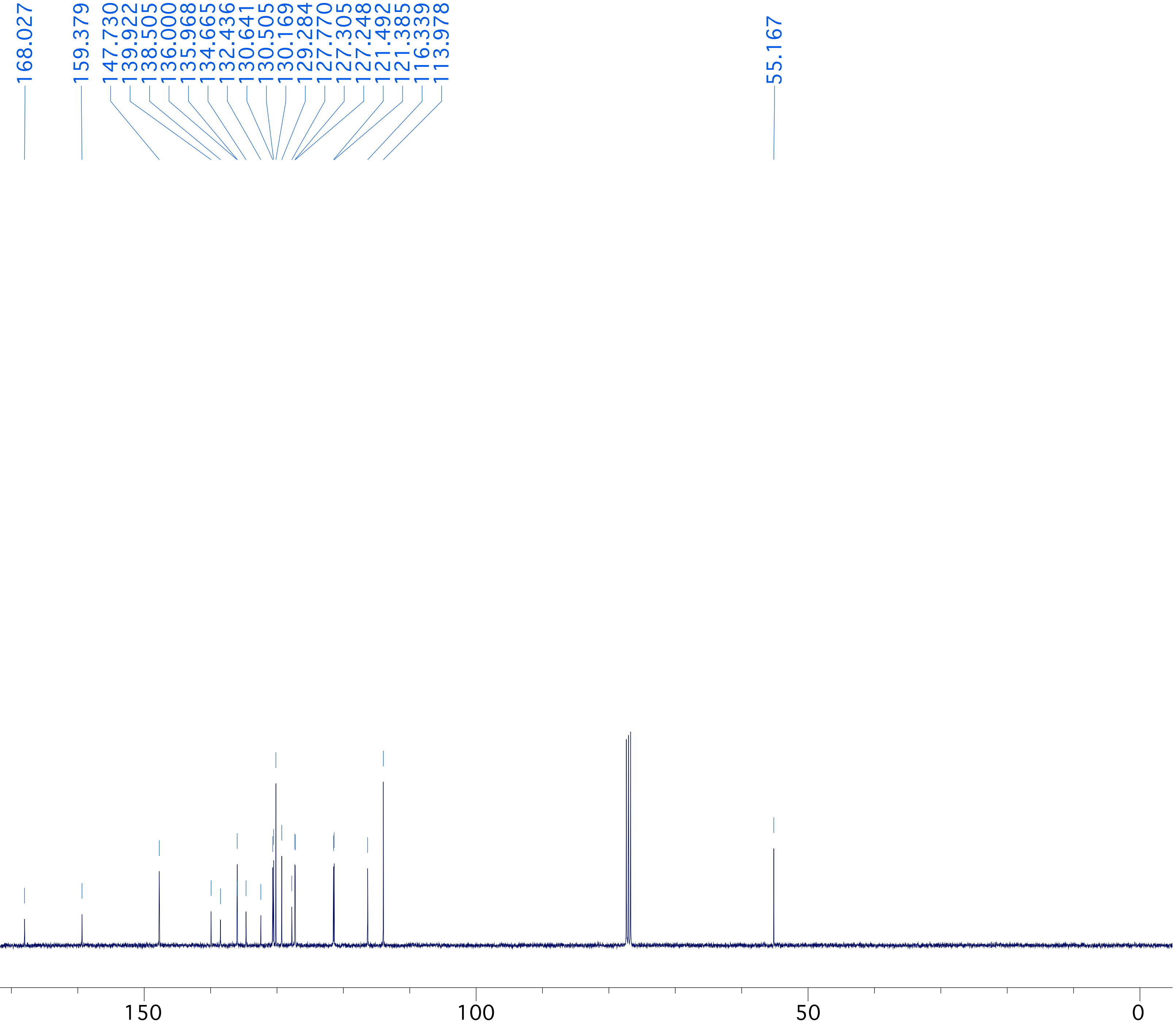

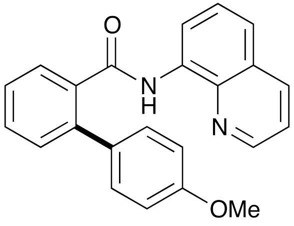

9a

^{1}H, $CDCl_3$, 400 MHz

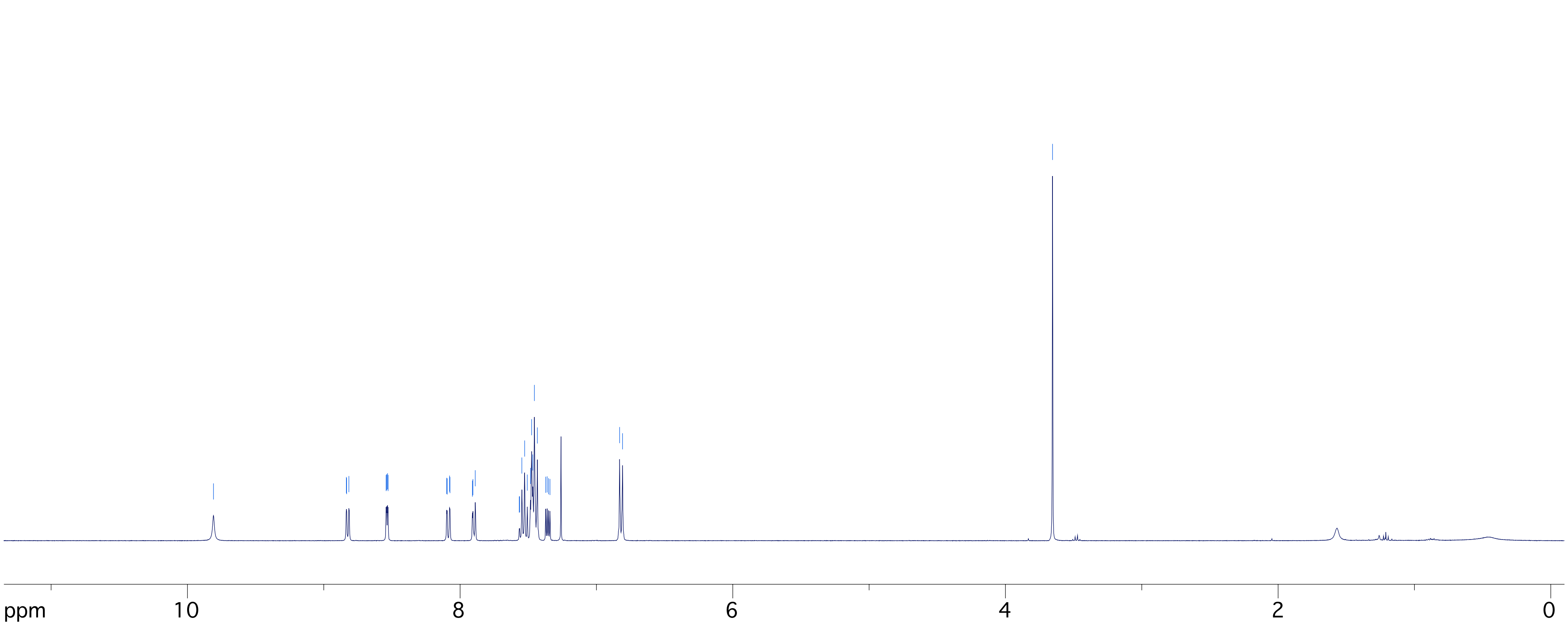

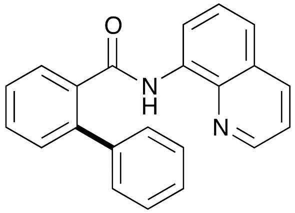

9b

^{13}C, $CDCl_3$, 125 MHz

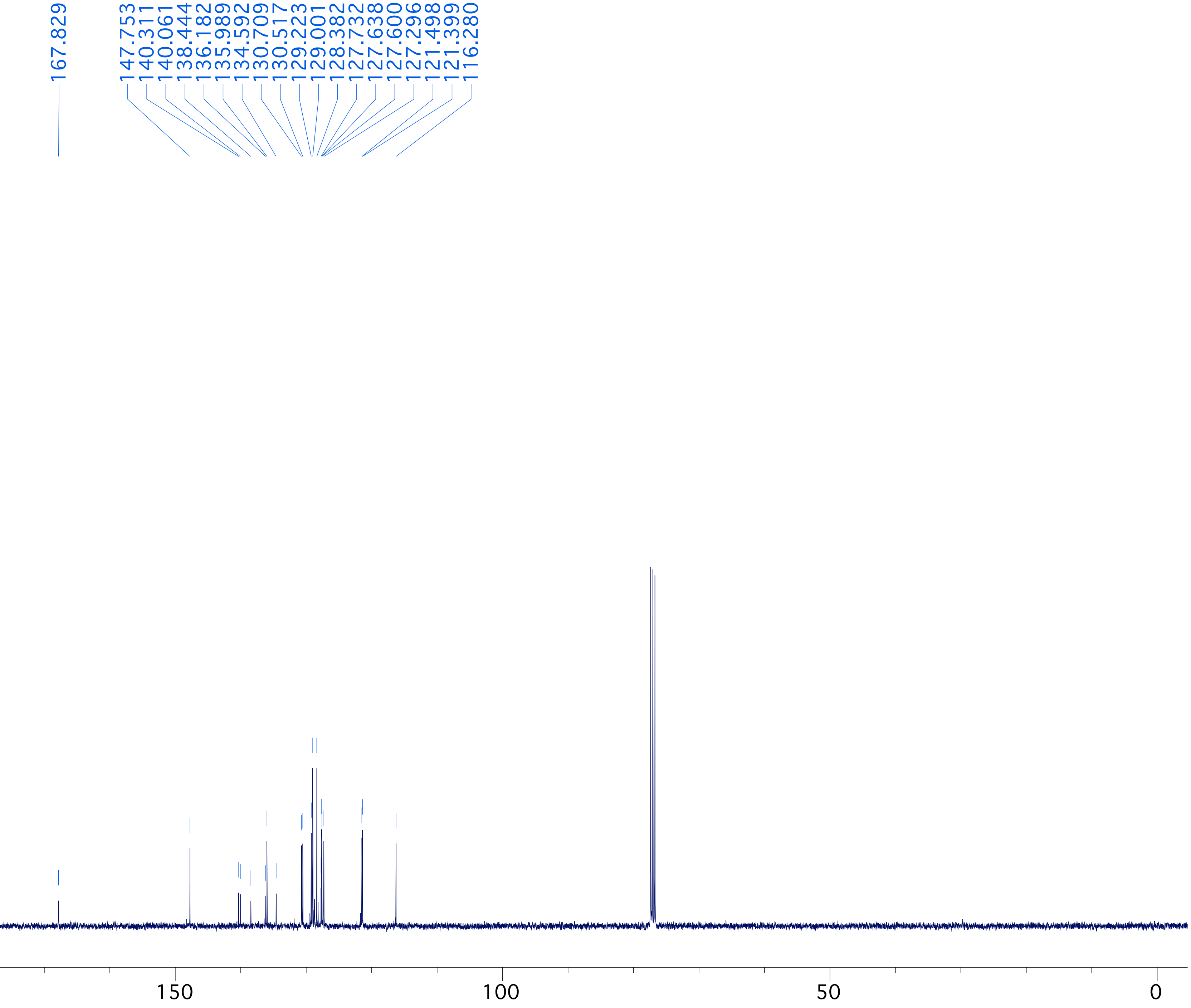

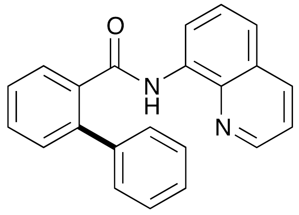

9b

^{1}H, $CDCl_3$, 400 MHz

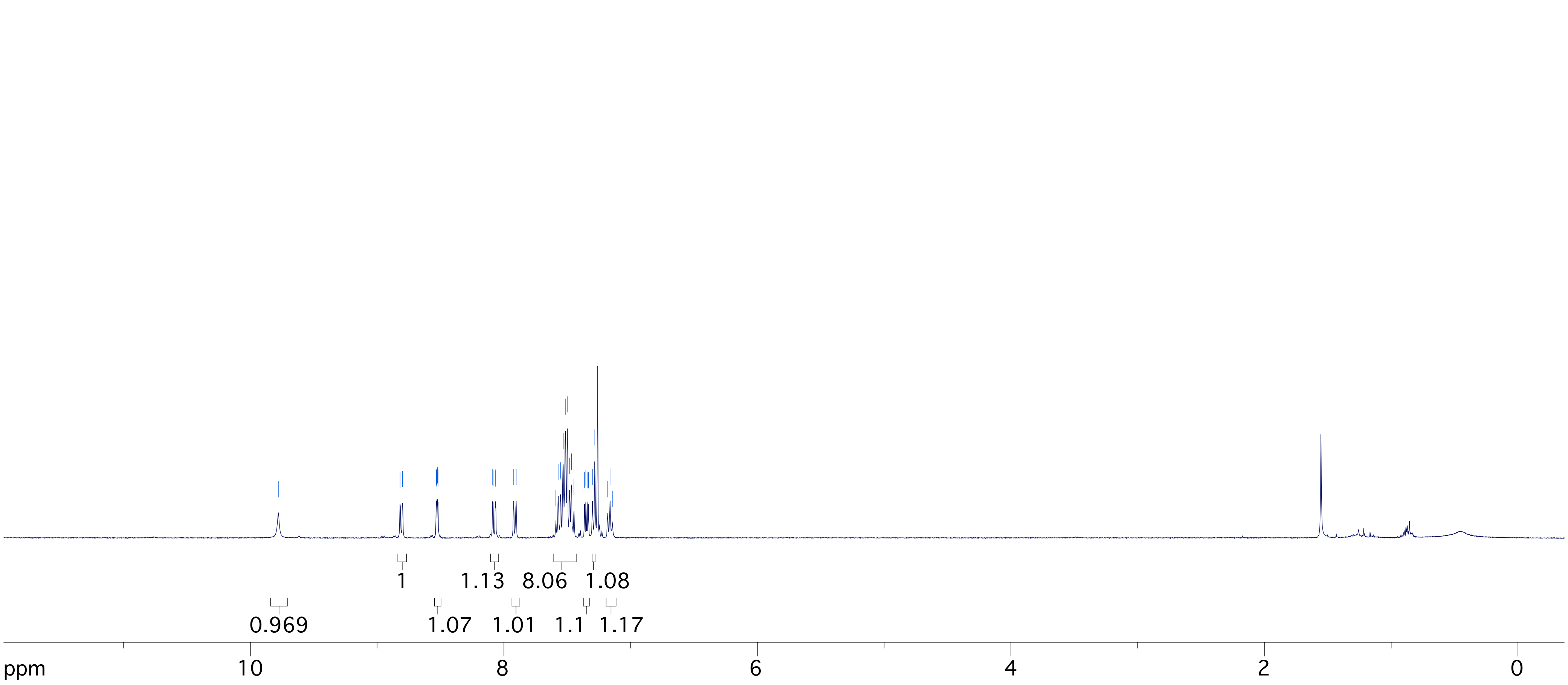

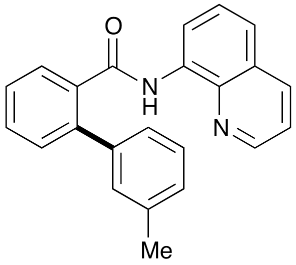

9c

^{13}C, $CDCl_3$, 125 MHz

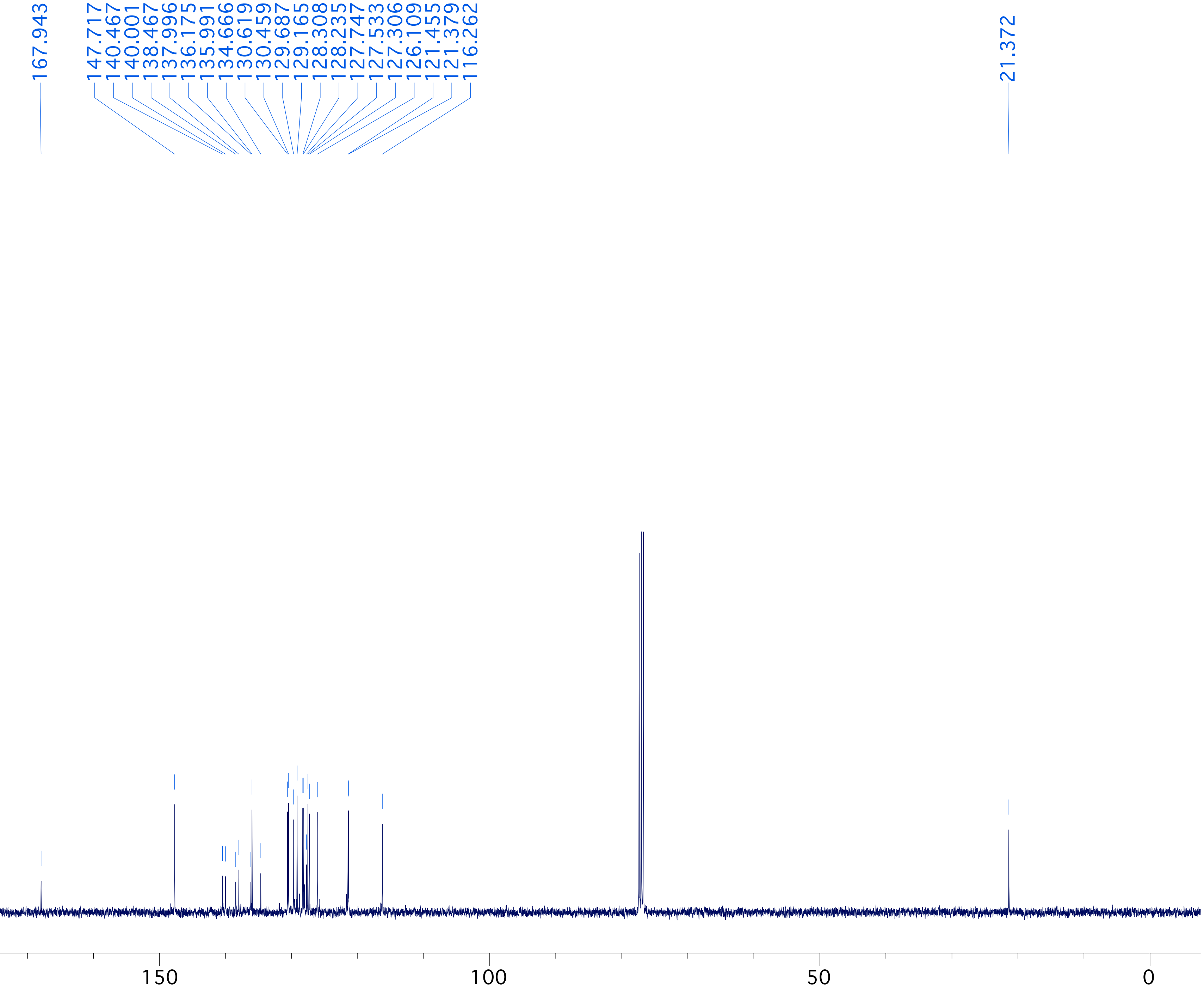

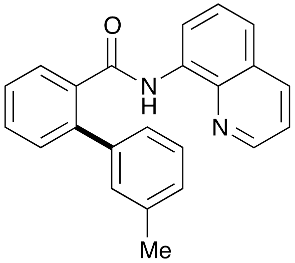

9c

^{1}H, $CDCl_3$, 400 MHz

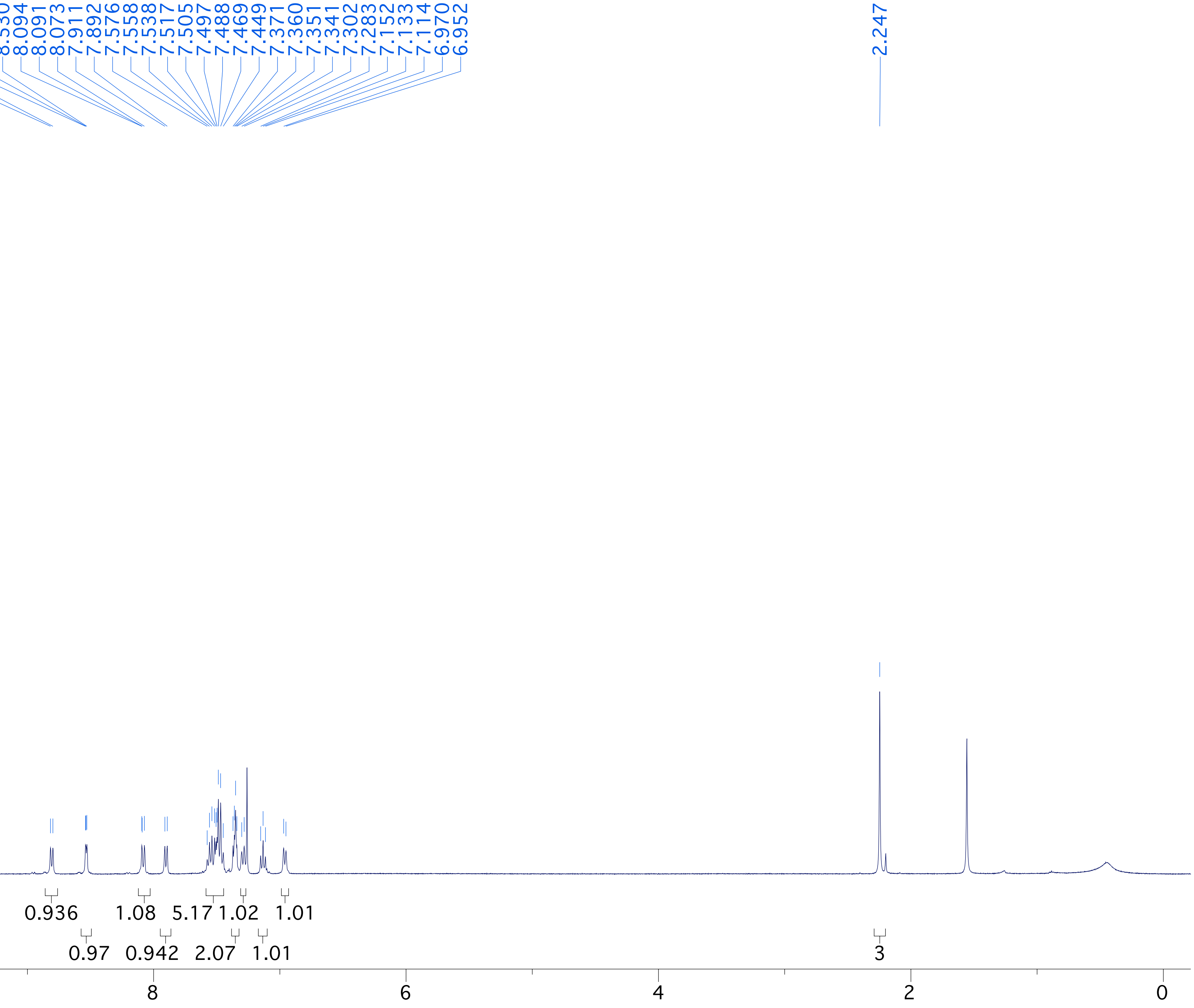

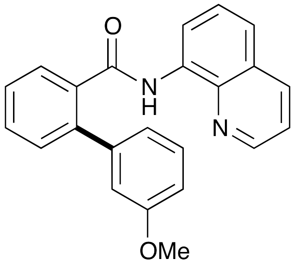

9d

^{13}C, d_3-MeCN, 125 MHz

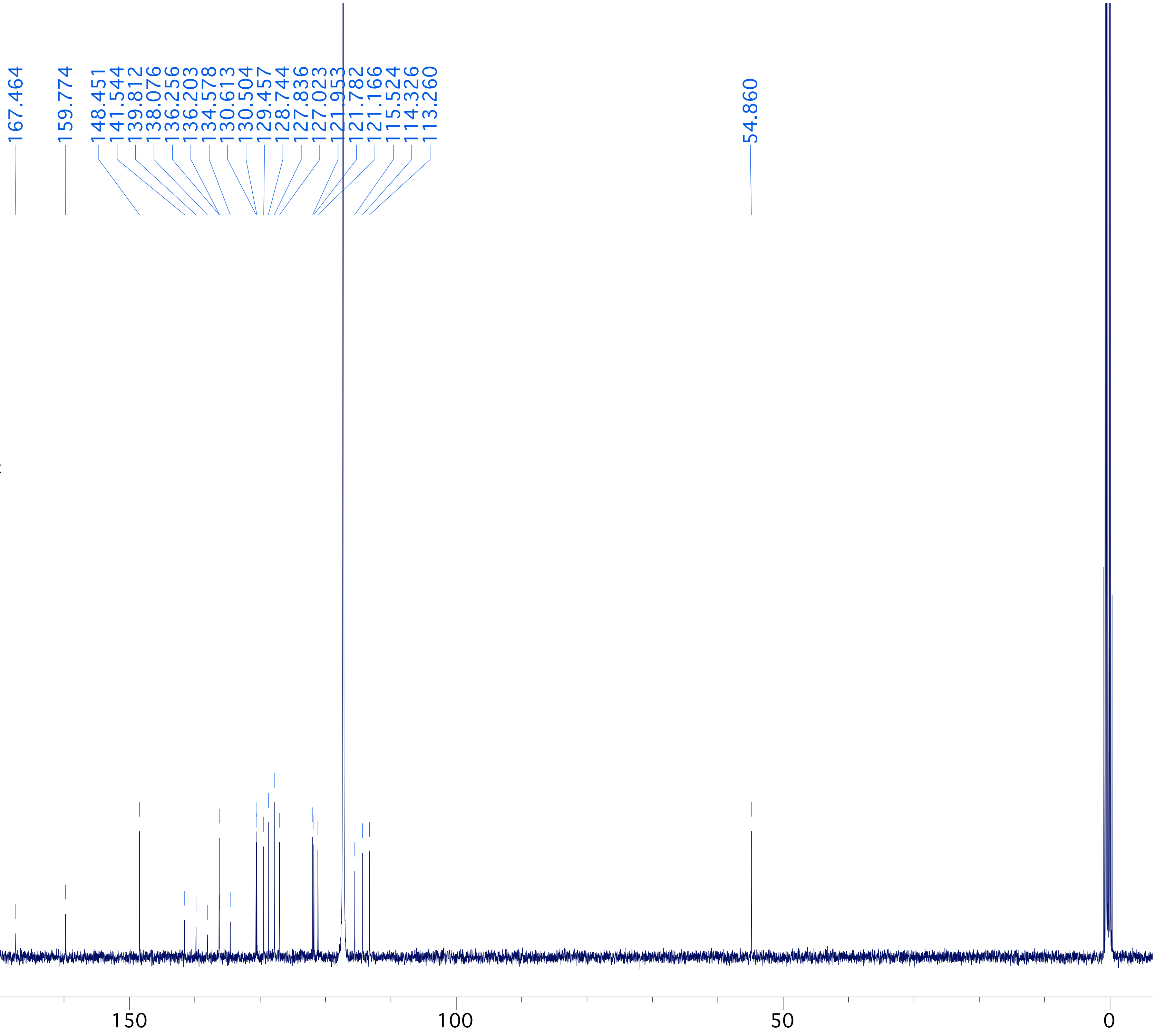

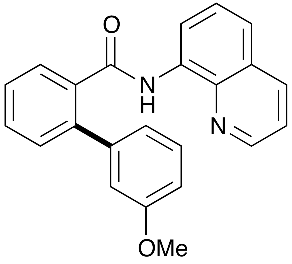

9d

^{1}H, $CDCl_3$, 400 MHz

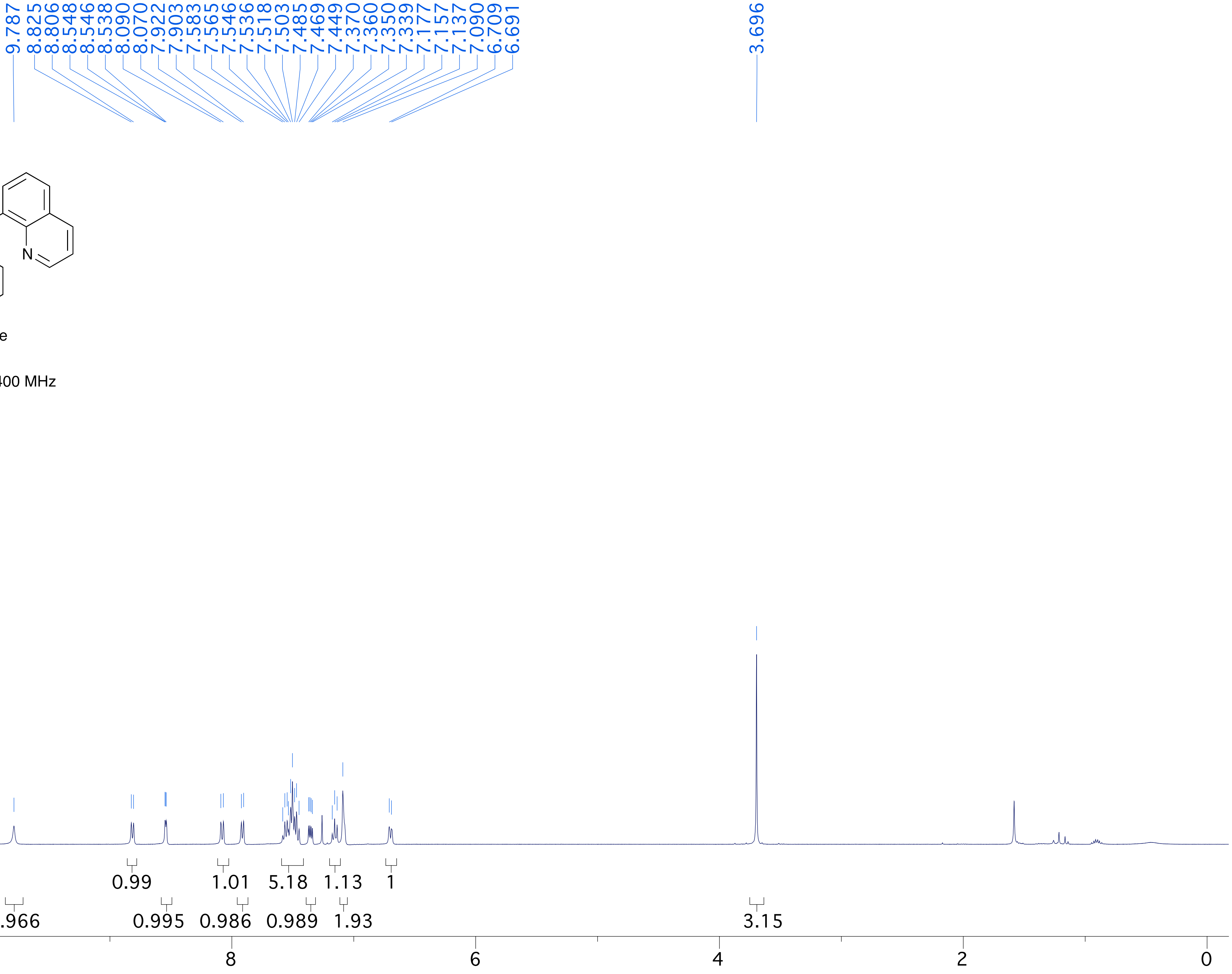

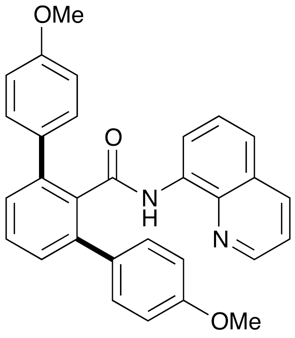

10a

^{13}C, $CDCl_3$, 125 MHz

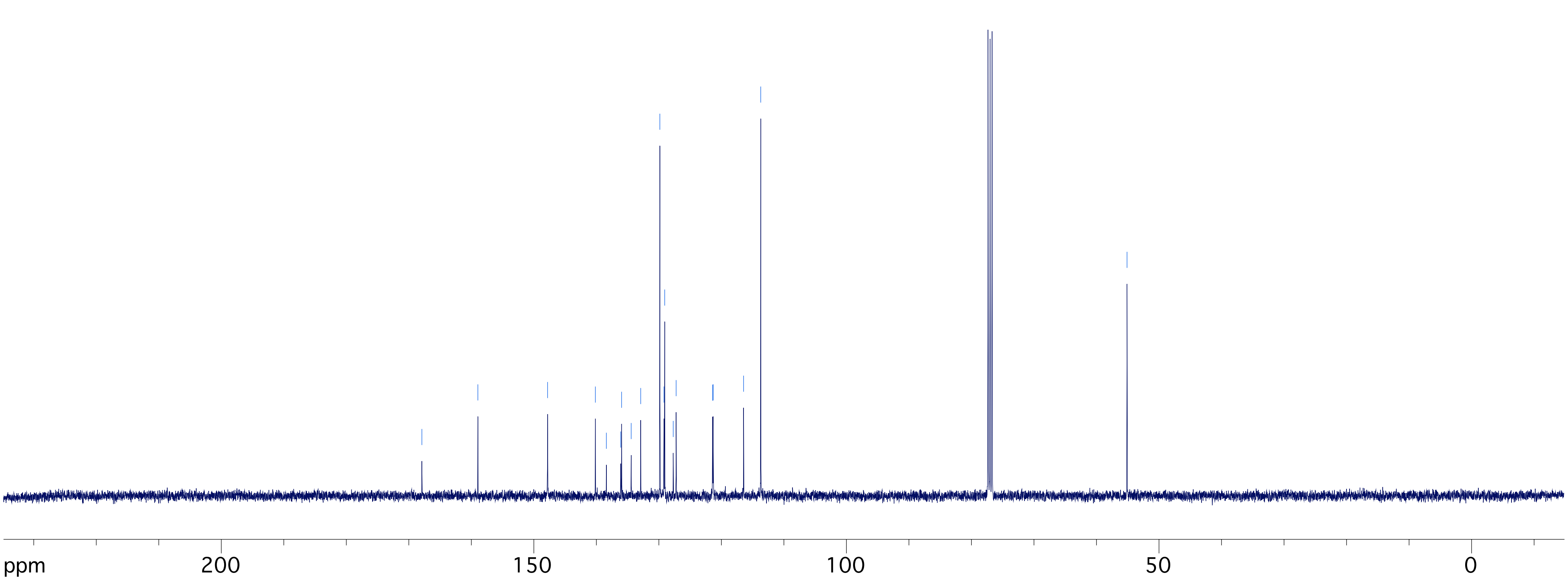

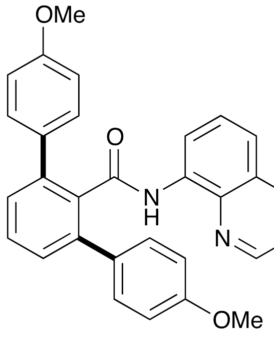

10a

^1H, $CDCl_3$, 400 MHz

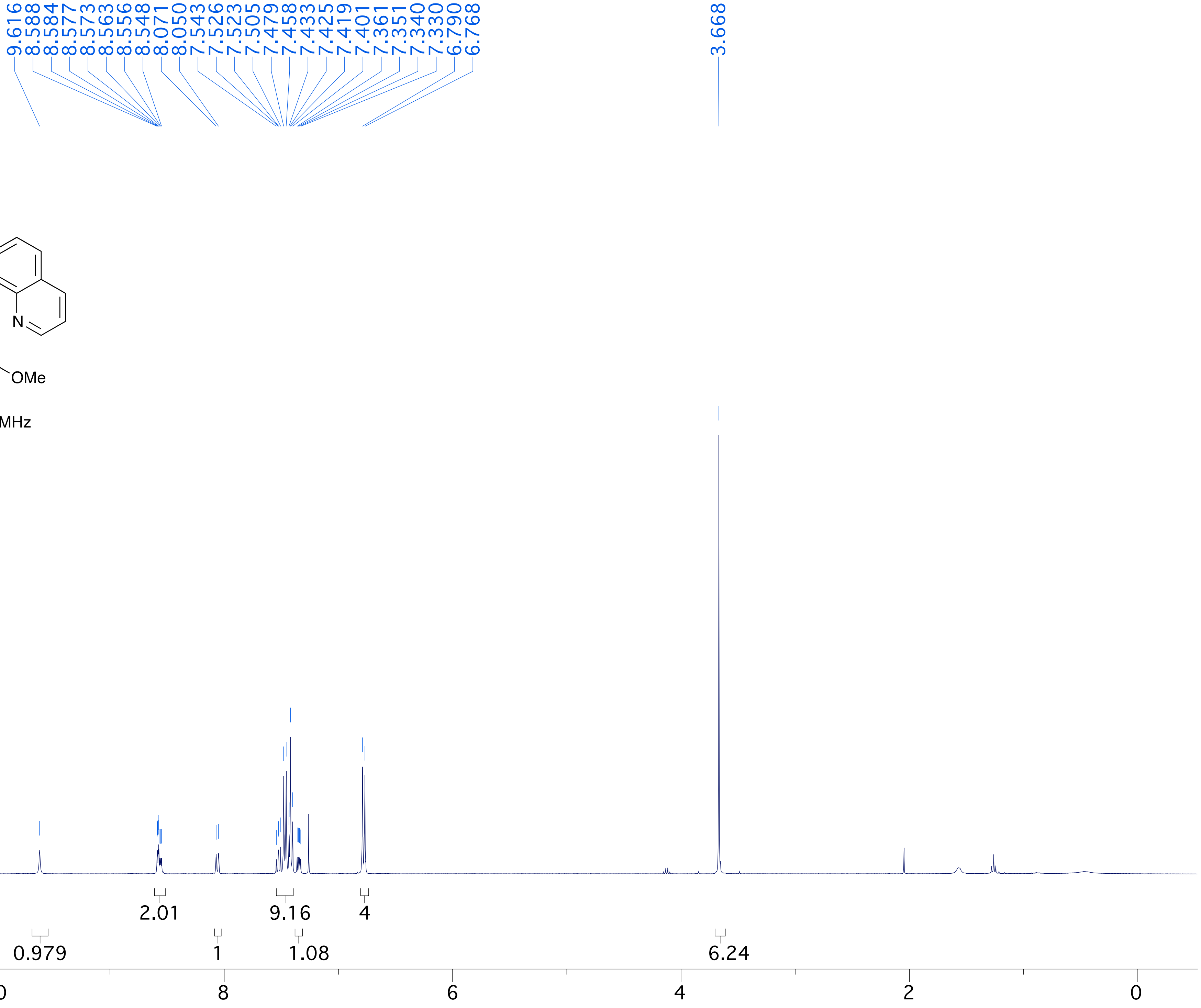

Supplement: Supplementary file 1 [file SC-006-C5SC00268K-s001.pdf]
